# The Role of Sphingolipids in Myocardial Recovery Mediated by Mechanical Unloading and Circulatory Support

**DOI:** 10.1016/j.jacbts.2025.101435

**Published:** 2025-12-19

**Authors:** Rana Hamouche, Sean M. Tatum, Elizabeth Dranow, J. Alan Maschek, Christos P. Kyriakopoulos, Thirupura S. Shankar, Joseph R. Visker, Jing Ling, Konstantinos Sideris, Craig H. Selzman, Abdallah G. Kfoury, Josef Stehlik, Rami Alharethi, James C. Fang, TingTing Hong, Sutip Navankasattusas, Matthew T. Rondina, William L. Holland, Scott A. Summers, Stavros G. Drakos, Eleni Tseliou

**Affiliations:** a Utah Cardiac Recovery (UCAR) Program, Salt Lake City, Utah, USA; b Nora Eccles Harrison Cardiovascular Research and Training Institute, University of Utah, Salt Lake City, Utah, USA; c Department of Nutrition and Integrative Physiology and the Diabetes Metabolism Research Center, University of Utah, Salt Lake City, Utah, USA; d Department of Pharmacology and Toxicology, College of Pharmacy, University of Utah, Salt Lake City, Utah, USA; e University of Utah Molecular Medicine Program, Eccles Institute of Human Genetics, Salt Lake City, Utah, USA; f Department of Internal Medicine, University of Utah Health, Salt Lake City, Utah, USA; g George E. Wahlen Veterans Affairs Medical Center and Geriatric Research Education and Clinical Center, Salt Lake City, Utah, USA

**Keywords:** ceramides, heart failure, myocardial recovery, sphingolipids, sphingosine-1-phosphate

## Abstract

Myocardial recovery after left ventricular assist device (LVAD) support is a critical phenomenon that allows advanced heart failure patients to retain their native heart. We quantified targeted sphingolipids in serum and cardiac tissue of patients who exhibited recovery post-LVAD. Our analysis revealed sustained elevated circulating ceramides levels in nonresponders post-LVAD, whereas responders showed reduced sphingosine-1-phosphate (S1P) levels. In contrast, cardiac tissue from nonresponders displayed increased S1P levels. We suggest that diminished ceramide and S1P may contribute to an increased likelihood of recovery after LVAD support. Collectively, our findings implicate the sphingolipid metabolic pathway as a potential therapeutic target to promote myocardial recovery after mechanical support.

## Highlights

• The circulating and cardiac sphingolipid profiles in patients showing myocardial recovery following LVAD support are described.
• Circulating dhCer(16:0) associated with functional and structural changes in advanced HF following LVAD support.
• We identify circulating and cardiac Cer and S1P as potential therapeutic targets for myocardial recovery.

## Visual Abstract

**Figure:**
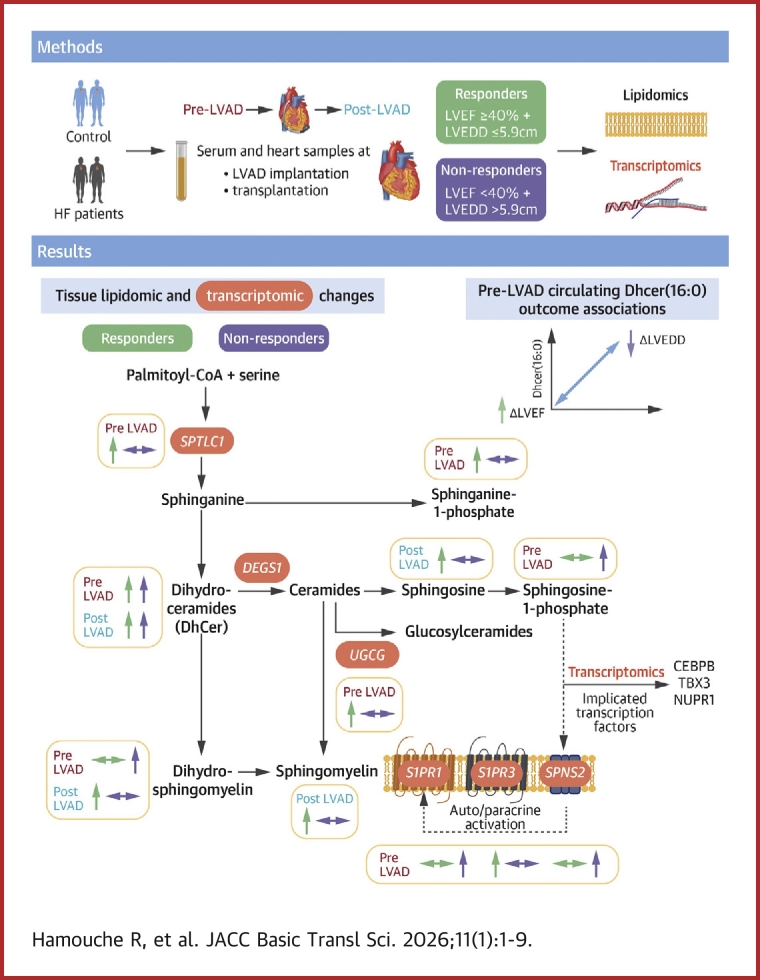

Heart failure (HF) remains the leading cause of death worldwide.^1^ Whereas heart transplantation is an established treatment for advanced HF, the limited availability of donor hearts has led to the use of left ventricular assist devices (LVADs) as an alternative bridge to transplant.^2^ Based on previous studies, 10%-30% of patients with advanced HF who undergo LVAD support improve significantly their cardiac function and structure following optimal HF pharmacologic therapy combined with 2 of the major pathophysiological changes mediated by LVAD support. First, the LVAD removes a significant part of the excess load and myocardial wall stress (ie, mechanical unloading) that is known to drive part of the adverse cardiac remodeling^3^ and second, LVAD supports the circulation and improves the systemic perfusion and the neurohormonal milieu.^4^, ^5^, ^6^, ^7^, ^8^, ^9^ These clinical improvements are associated with mechanistic changes described by molecular studies of human myocardial samples obtained before and after LVAD support, which showed partial reversal of several components of cardiac remodeling including cardiomyocyte hypertrophy and biology, extracellular matrix, microvasculature, myocardial metabolism, and other.^10^^,^^11^

Under normal physiological conditions, cardiomyocytes are metabolically adaptable, using various substrates such as fatty acids (FAs), carbohydrates, and amino acids to meet the high energy demands to contractile function and blood flow.^12^ In HF, cardiomyocytes lose their metabolic flexibility and are unable to meet metabolic demand through oxidative phosphorylation.^13^ This alteration in energetic fuel synthesis and use is coupled with the production of biosynthetic precursors and the accumulation of toxic lipid intermediates such as sphingolipids (SLs).^14^^,^^15^

SLs are a significant class of bioactive lipids typically characterized by a palmitic acid–derived sphingoid base containing an amino alcohol headgroup and a variable FA carbon side chain. They serve essential structural roles in cellular membranes and their metabolites play an important role in various cellular processes, including cell signaling, apoptosis, and inflammation.^16^, ^17^, ^18^ Dysregulation of SL metabolism has been implicated in the pathophysiological mechanisms of various diseases, including insulin resistance, obesity, and HF.^19^^,^^20^ Enzymes involved in SL metabolism are becoming potential therapeutic targets in various neurologic and oncologic diseases.^21^^,^^22^ Among these lipid species, ceramides (Cer) have received attention for their association with lipotoxic cardiomyopathy, heart cell survival, cardiac remodeling, and inflammatory responses.^23^^,^^24^ In cardiomyocytes, Cer are produced through 2 different pathways: the de novo pathway and the sphingomyelinase pathway.^20^^,^^25^^,^^26^ Through the de novo pathway, Cer synthases (CERS), a family of 6 synthases, attach FAs of varying chain lengths to the sphinganine backbone forming Cer with distinct chain lengths that serve diverse cellular and signaling functions.^27^ Cer then follow one of the catalytic pathways forming sphingosine and sphingosine-1-phosphate (S1P), glucosylceramides (GlcCer), or sphingomyelins (SMs)^28^^,^^29^ (Figure 1). Previous studies have shown that patients with HF have increased levels of Cer(16:0), Cer(16:1), and Cer(24:1) in cardiac tissue,^20^ whereas systemic administration of S1P has indicated cardioprotective effects after ischemia in murine models,^30^^,^^31^ showcasing the significant role of SLs in cardiac toxicity and biosignaling.^32^, ^33^, ^34^ However, the causes and consequences of these lipid alterations are incompletely understood and the changes in SL levels in advanced HF patients before and after LVAD support and their potential contribution to myocardial recovery post-LVAD support have not been studied. We hypothesized that HF disrupts systemic and cardiac lipid metabolism leading to changes in SL composition and that LVAD support followed by myocardial recovery may modify the SL profile.

**Figure 1:**
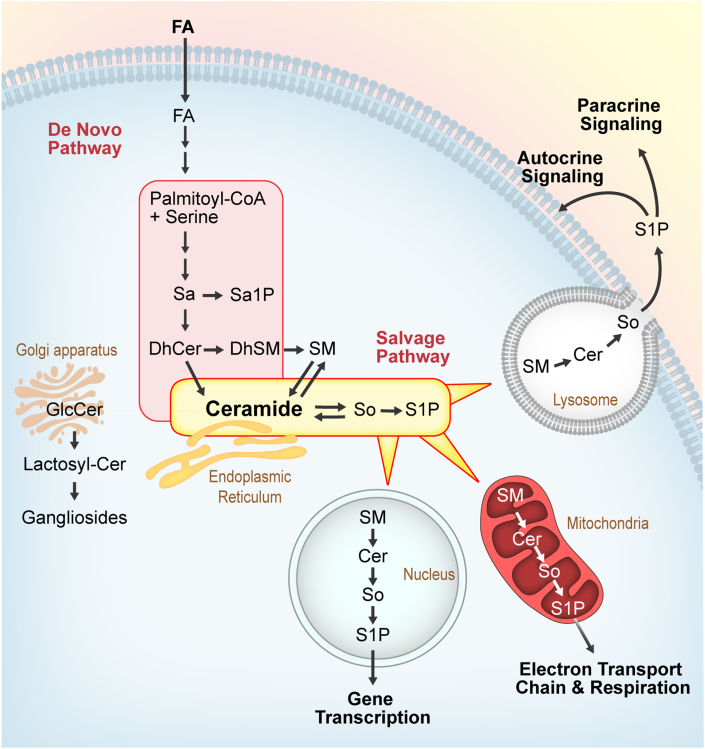
SL Metabolism in Cardiomyocytes and the Multifaceted Roles of S1P in Cellular Signaling Ceramides are produced through the de novo and the salvage pathways and give rise to SM, GlcCer and So/S1P. These metabolites function to regulate gene transcription, mitochondrial respiration and signaling. Cer = ceramides; CoA = coenzyme A; dhCer = dihydroceramides; dhSM = dihydrosphingomyelins; FA = fatty acid; GlcCer = glucosylceramides; S1P = sphingosine-1-phospate; Sa = sphinganine; Sa1P = sphongosine-1-phosphate; SL = sphingolipid; SM = sphingomyelin; So = sphingosine.

## Methods

### Study population

Consecutive advanced HF patients receiving a continuous-flow LVAD between May 2008 and June 2019 at one of the institutions comprising the Utah Cardiac Recovery Program (University of Utah Health and School of Medicine, Intermountain Medical Center, and George E. Wahlen Department of Veterans Affairs Medical Center) were included. Patients were followed until LVAD explantation due to heart transplantation or cardiac recovery, loss to follow-up, or death. Patients were excluded if they were previously diagnosed with hypertrophic or infiltrative cardiomyopathies, had a baseline left ventricular ejection fraction (LVEF) ≥40%, withdrew consent, or had inadequate (<3 months) post-LVAD follow-up (early heart transplantation, death, or unavailable echocardiographic follow-up). Patients with unavailable serum and/or tissue samples at either pre-LVAD implantation or post-LVAD support time points were excluded.

### Ethical approval for human samples

Blood samples and apical cardiac tissue samples from advanced HF patients were used in this study. Control tissue samples were acquired from organ donors with normal cardiac function based on echocardiography data that were rejected for transplant for noncardiac reasons. Control blood samples were acquired from healthy, medication-free, ambulatory donors. The Institutional Review Board (protocol number 00095106) of each of the participating institutions approved the study and all patients and healthy donors provided informed consent.

### Clinical management and definitions

Data collection included demographics, medications, laboratory values prior to and closest to the LVAD implantation. Cardiac imaging data were obtained before and during LVAD support to assess the structural and functional effects of mechanical support on the failing heart. The duration of HF was defined as the time from HF symptom onset to LVAD implantation as ascertained through chart review and, if the documentation was unclear, through direct patient documentation when possible.

The effect of LVAD support on cardiac size, shape, and function was assessed by echocardiography and invasive hemodynamic measurements following LVAD implantation and prior to discharge as previously described.^11^ LVAD speed was adjusted to: 1) achieve optimal unloading of both the left and right ventricles (ie, intraventricular septum in the middle); 2) minimize mitral regurgitation; and 3) allow occasional aortic valve opening.^11^ Subsequent speed adjustments were made as indicated by patients’ symptoms and/or clinical events. Patients were medically managed at the discretion of the treating physicians within the participating institutions per established standard HF guideline-directed medical therapy.

### Echocardiography

Transthoracic echocardiograms were performed within the 2 weeks preceding, and serially at 1, 3, 6, 9, and 12 months following LVAD implantation as previously described.^11^ The standard of care clinical protocol entailed 2 sets of echocardiographic measurements: 1) at full LVAD support; and 2) after 30 minutes of limited support, at the lowest setting recommended by the device manufacturer. Complete echocardiographic assessment, including 2-dimensional, M-mode, and Doppler modalities, was performed according to current American Society of Echocardiography and European Association of Cardiovascular Imaging guidelines.^35^

To quantitatively estimate functional and structural cardiac improvement on LVAD support, we used the following formulas, respectively:

LVEF change (ΔLVEF) = LVEF post-LVAD − LVEF pre-LVAD

Left ventricular end-diastolic diameter (LVEDD) change (ΔLVEDD) = LVEDD post-LVAD − LVEDD pre-LVAD

For both LVEF and LVEDD pre-LVAD, we used the measurement prior to and closest to LVAD implantation, whereas for LVEF and LVEDD post-LVAD, we used the maximum LVEF and minimum LVEDD achieved within the 12-month period following LVAD implantation.

Based on the combined change in LV function and structure after LVAD support, the patients were categorized into 2 groups. We defined as responders patients who showed both functional and structural improvement (LVEF ≥40%, and LVEDD ≤5.9 cm) within 12 months post-LVAD implantation. Nonresponders comprised the remaining patients who did not meet criteria to be classified as responders.

### Targeted lipidomics

Lipid analysis was performed using a targeted tandem mass spectrometry (MS/MS) panel to quantify 62 specific lipid species across predefined SL classes, including dihydroceramides (dhCer), Cer, GlcCer, sphinganine, sphingosine, sphinganine-1-phosphate (Sa1P), S1P, dihydrosphingomyelins (dhSM), SMs, phosphatidylcholine (PC), diacylglycerol, and triacylglycerol.

#### Chemicals

Liquid chromatography (LC)-MS–grade solvents and mobile phase modifiers were obtained from Honeywell Burdick and Jackson (acetonitrile, isopropanol, formic acid), Thermo Fisher Scientific (methyl *tert*-butyl ether), and Sigma-Aldrich/Fluka (ammonium formate, ammonium acetate). Lipid standards were obtained from Avanti Polar Lipids.

#### Sample preparation

Serum lipids were extracted using a modified Alshehry protocol. Briefly, 20 μL of serum was mixed with 200 μL of butanol:methanol (1:1, vol/vol) containing internal standards (deuterated Cer, SMs, GlcCer, and S1P), then vortexed, sonicated, and centrifuged at 20 °C. The supernatant was transferred to LC-MS vials for analysis.^36^^,^^37^

Tissue lipids were extracted using a modified Matyash protocol. Tissues were homogenized in phosphate-buffered saline using a TissueLyser (Qiagen), and 188 μL of homogenate was combined with 225 μL methanol containing the same internal standards and 750 μL methyl tertiary butyl ether. After incubation on ice and phase separation by centrifugation, the upper phase was dried and reconstituted in methanol:toluene (9:1) for LC-MS analysis.^38^

### LC-MS analysis

Lipid extracts were analyzed using an Acquity UPLC CSH C18 column (2.1 × 100 mm, 1.7 μm; Waters Corp) with a VanGuard precolumn (2.1 × 5 mm, 1.7 μm; Waters Corp) on an Agilent 1290 Infinity LC system coupled to a 6490 triple quadrupole MS. Chromatography was performed at 65 °C with dynamic multiple reaction monitoring (dMRM) in positive ion mode. For general lipid analysis, mobile phase A was ACN:H_2_O (60:40, vol/vol) with 10 mmol/L ammonium formate and 0.1% formic acid; mobile phase B was IPA:ACN:H_2_O (90:9:1, vol/vol/vol) with the same additives. The gradient (flow rate 0.4 mL/min) ramped from 15% to 99% B over 7.14 minutes, held to 9.45 minutes, and returned to initial conditions at 9.8 minutes. Injection volume was 3 μL; samples were randomized with pooled quality control (QCs) injected 8 times. dMRM transitions included [M+H]^+^→284.3 for dhCer, 264.2 for Cer, 271.3 for labeled Cer; [M+H]^+^→184.1 for SM and PC; and [M+NH_4_]^+^→ neutral loss of acyl chains for glycerolipids. Lipids without standards were identified via high-resolution LC/MS based on retention time and diagnostic ions.

Sphingoid base phosphates were analyzed using the same LC-MS instrumentation and column configuration. The only difference was the chromatographic gradient: mobile phase A was MeOH:H_2_O (58:42, vol/vol) with 0.7% formic acid, and B was MeOH:IPA:H_2_O (90:8:1, vol/vol/vol) with 0.7% formic acid. The gradient (flow rate 0.5 mL/min) started at 50% B, held for 1 minute, ramped to 99% by 5.4 minutes, held to 7.9 minutes, and returned to 50% at 8 minutes. Injection volume was 10 μL; samples were analyzed in randomized order. dMRM transitions included [M+H]^+^→284.3 for Sa1P and Sa, 264.2 and 271.2 for S1P and S1P-d7, and 282.3 for sphingosine.

### LC-MS data processing

Raw data were processed using Agilent MassHunter Workstation, including the Qualitative and Quantitative Analysis modules. Pooled QC samples (n = 8) and process blanks (n = 4) were injected throughout the run to monitor data quality. Lipid peak areas were exported from MassHunter Quantitative and evaluated in Excel (Microsoft). Lipids were retained for analysis only if their relative SD in QC samples was <30% and their signal in process blanks was <30% of the corresponding QC average. Data were normalized to class-specific internal standards and then to sample amount.

### RNA isolation, sequencing, and bioinformatics

Total RNA was isolated from 50 mg of frozen human myocardial LV tissue samples using the miRNeasy Mini Kit (Qiagen). To remove all DNA, an on-column RNase-free DNase digestion was used. All samples were processed at the same time for dispersion estimation and normalization, with contrasts applied to specific condition groups for differential expression testing. A batch factor was introduced in the model to account for different sequencing batches. The human GRCh38 genome and gene annotation files were downloaded from Ensembl (release 87). Reads were trimmed of adapters using cutadapt 1.16^39^ and aligned to the reference database using the STAR (version 2.5.4a).^40^ Mapped reads were assigned to annotated genes using featureCounts (version 1.6.3).^41^ Differentially expressed genes were identified by comparing 20 pre-nonresponder vs 9 preresponder samples using a 10% false discovery rate with DESeq2 (version 1.42.1).^42^ The 222 significant genes were loaded into ingenuity pathway analysis to discover enriched pathways and upstream regulators Activated and inhibited pathways were further identified by checking activation z-scores^43^ with absolute values >2.

### Statistical analysis

Patient baseline characteristics are presented using standard summary statistics including frequencies, percentages, and means. Measures of variation are presented as the mean ± SD, or median (Q1-Q3) as appropriate. Differences between patient groups for categorical variables were evaluated using the chi-square test or Fisher exact test as appropriate, and continuous variables were evaluated using 2-group Student’s *t*-tests or Wilcoxon rank-sum tests as appropriate. Assumptions of normality were assessed graphically with histograms and Q-Q plots.

Comparisons of all SL serum data for control and patients included in pre-LVAD and post-LVAD analyses were performed using a mixed-model approach, with response status and time (pre-/post-LVAD) as fixed effects, and the patient identifier as a random effect, allowing us to include patients for whom we had measures both pre- and post-LVAD as well as patients for whom we had measures at only 1 time point. Comparisons of tissue SLs among control, nonresponder, and responder groups were performed using 1-way analysis of variance. Significant analyses of variance were followed by Sidak-adjusted post hoc pairwise tests.^44^ Adjustments for multiple comparisons of all SL serum data were performed using the Benjamini-Krieger-Yekutieli false discovery rate approach.^45^ Associations between pre-LVAD SLs and clinical characteristics were assessed using linear regressions. Statistical analyses were performed using Stata (version 18.5, StataCorp). A 2-sided *P* value <0.05 was considered statistically significant.

## Results

### Demographic data in responders and non-responders at time of LVAD implantation

**Figure 2:**
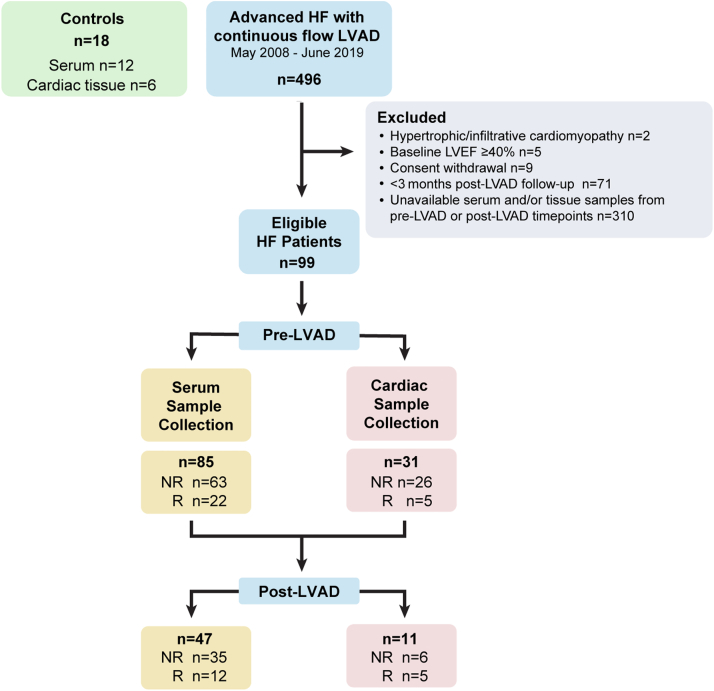
Patient Selection Flowchart and Exclusion Criteria Responders are defined as patients who achieved a left ventricular ejection fraction (LVEF) ≥40% and LV end-diastolic diameter ≤5.9 cm after 12 months of left ventricular assist device (LVAD) support. Nonresponders (NR) are described as the rest of the patients. Control tissues were acquired from organ donors with normal cardiac function based on echocardiography data that were rejected for transplant for noncardiac reasons, whereas control blood samples were acquired from healthy, medication-free, ambulatory donors. HF = heart failure; R = responders.

There were 99 patients that met our inclusion criteria. Of these, hypothesis-driven lipid analysis was performed on serum samples from 85 HF patients at time of LVAD implantation and 12 control subjects (Figure 2). Baseline demographic, laboratory, and echocardiographic data are presented in Table 1. Compared to nonresponders, responders were more likely to be female (31.8% vs 8.2%; *P* = 0.006), have a shorter duration of HF (median: 10 [Q1-Q3: 1-31] vs 74 [Q1-Q3: 48-126] months; *P* < 0.001), and reduced LVEDD (6.91 ± 1.16 vs 6.42 ± 0.96 cm; *P* = 0.079). There were no significant differences between the 2 groups in prior medical history, etiology of HF, preoperative therapies including inotropes, and temporary mechanical circulatory support use (Table 1). With regard to guideline-directed medical therapy, there were no differences between the 2 groups. Responders were less likely to be on statins (9.1% vs 49.2%; *P* = 0.001) than nonresponders were at the time of the LVAD implantation. We did not observe any differences in responders and nonresponders in blood glucose (107.1 ± 23.2 vs 124.1 ± 40.3 g/dL; *P* = 0.38), HbA_1c_ (6.36% ± 1.04% vs 5.97% ± 0.95%; *P* = 0.28), low-density lipoprotein (81.3 ± 39.5 vs 86.2 ± 20.5 mg/dL; *P* = 0.79), high-density lipoprotein (29.8 ± 9.4 vs 33.2 ± 9.1 mg/dL; *P* = 0.46), and triglyceride levels (123.8 ± 69.8 vs 96.0 ± 48.7 mg/dL; *P* = 0.39). Echocardiographic parameters including LVEF (18.7% ± 6.8% vs 20.4% ± 8.8%; *P* = 0.36) and LV end-systolic diameter (6.32 ± 1.16 vs 6.42 ± 8.8 cm; *P* = 0.15) did not differ between the 2 groups.

**Table 1:** Baseline Demographic, Clinical Characteristics, Echocardiographic and Laboratory Characteristics in Serum Cohort: Control, NR, and R Groups

	Total Cohort (N = 97)	Control Subjects (n = 12)	Nonresponders (n = 63)	Responders (n = 22)
Male	81 (83.5)	8 (66.7)	58 (92.0)^a^	15 (68.2)
Age, y	56.6 ± 14.3	53.3 ± 12.9	57.8 ± 13.1	55.1 ± 18.3
Race				
Native American	2 (2.0)	—	2 (3.3)	0 (0)
Black or African American	5 (5.2)	—	3 (5.0)	2 (14.3)
Caucasian	77 (89.5)	12 (100)	55 (91.7)^a^	10 (71.4)
Other	2 (2.0)	—	0 (0)	2 (14.3)
Ethnicity				
Hispanic or Latino	5 (5.8)	0 (0)	3 (5.0)	2 (14.3)
Body mass index, kg/m^2^	28.6 ± 5.8	26.6 ± 4.1	29.0 ± 6.2	28.5 ± 5.0
Medical history				
History of hypertension	43 (50.6)	—	30 (47.6)	13 (59.1)
Diabetes mellitus	29 (34.5)	—	23 (37.1)	6 (27.3)
Atrial fibrillation	33 (39.3)	—	26 (41.3)	7 (33.3)
Previous thoracotomy	25 (29.4)	—	22 (34.9)	3 (13.6)
History of smoking	36 (37.1)	0 (0)^b^^,^^c^	26 (41.3)	10 (45.5)
History of ethanol use	30 (34.5)	0 (0)^b^^,^^c^	23 (38.3)	7 (46.7)
History of substance abuse	8 (8.3)	0 (0)	4 (6.4)	4 (18.2)
Preoperative supportive therapies				
Inotrope dependency	53 (54.6)	—	40 (63.5)	13 (59.1)
Intra-aortic balloon pump	7 (7.2)	—	7 (11.7)	0 (0)
Impella/VA-ECMO	4 (4.1)	—	2 (3.2)	2 (9.1)
NYHA functional class IV	55 (56.7)	—	44 (73.3)	11 (73.3)
Heart failure duration, mo	66 (18-120)	—	74 (48-126)^a^	10 (1-31)
Heart failure etiology				
Ischemic cardiomyopathy	32 (32.9)	—	25 (39.7)	7 (31.8)
Heart failure medications				
Beta blocker	61 (62.8)	—	43 (68.3)	18 (81.8)
ARB/ACE inhibitor	50 (51.5)	—	36 (57.1)	14 (63.6)
Aldosterone antagonist	50 (51.5)	—	40 (63.4)	10 (45.5)
Diuretic	82 (84.5)	—	62 (98.4)	20 (90.9)
Lipid-lowering medications				
Statins	33 (34.0)	—	31 (49.2)^a^	2 (9.1)
VAD type				
HeartMate 2	43 (44.3)	—	26 (41.3)^a^	17 (77.3)
HeartMate 3	4 (4.1)	—	3 (4.8)	1 (4.5)
Heartware	31 (27.0)	—	27 (42.8)	4 (18.2)
Jarvik	6 (6.1)	—	6 (9.5)	0 (0)
Levacor	1 (1.0)	—	1 (1.6)	0 (0)
Echocardiographic measurements				
Left ventricular ejection fraction, %	19.2 ± 7.3	—	18.7 ± 6.8	20.4 ± 8.8
Left ventricular end-diastolic diameter, cm	6.79 ± 1.13	—	6.91 ± 1.16	6.42 ± 0.96
Left ventricular end-systolic diameter, cm	6.22 ± 1.23	—	6.32 ± 1.23	5.79 ± 1.23
Laboratory measurements				
White blood cell count, ×10^3^/μL	8.82 ± 3.79	5.23 ± 0.87^b^	9.2 ± 4.0	8.7 ± 2.9
Hemoglobin, g/dL	12.6 ± 2.1	14.5 ± 1.0	12.5 ± 2.1	12.2 ± 2.0
Platelet count, ×10^3^/μL	210.4 ± 78.4	266.3 ± 55.6	199.0 ± 77.4	233.7 ± 79.1
Sodium, mEq/L	134.7 ± 5.4	141.3 ± 3.7^b^	134.1 ± 5.1	134.5 ± 5.3
Potassium, mEq/L	4.07 ± 0.46	4.38 ± 0.25	4.04 ± 0.50	4.03 ± 0.30
Blood urea nitrogen, mg/dL	—	—	29.4 ± 11.7	25.7 ± 17.3
Creatinine, mg/dL	1.50 ± 1.72	3.43 ± 6.16^b^	1.32 ± 0.44	1.45 ± 0.87
Blood glucose, g/dL	117.9 ± 38.3	82.7 ± 7.2^b^	124.1 ± 40.3	107.1 ± 23.2
Uric acid, mg/dL	8.1 ± 2.5	—	8.4 ± 2.4	7.1 ± 2.6
Aspartate aminotransferase, mg/dL	31.0 (22.0-43.0)	22.5 (22.0-23.0)^b^^,^^c^	32 (23-44)	35 (24-49)
Alanine aminotransferase, mg/dL	27.0 (21.0-51.0)	18 (12-32)	27 (22-51)	32 (18-62)
Alkaline phosphatase, mg/dL	93.0 (70.0-118.0)	62.5 (58.0-68.0)^b^^,^^c^	93 (71-119)	106 (85-128)
Total serum protein, g/dL	7.02 ± 0.82	7.07 ± 0.23	6.9 ± 0.8	7.3 ± 0.9
Albumin, g/dL	3.87 ± 0.53	4.58 ± 0.28^b^^,^^c^	3.81 ± 0.52	3.85 ± 0.42
Total bilirubin, mg/dL	—	—	1.49 ± 1.08	1.41 ± 1.87
Direct bilirubin, mg/dL	—	—	1.05 ± 1.07	0.45 ± 0.39
International Normalized Ratio	1.30 ± 0.39	0.97 ± 0.05	1.33 ± 0.36	1.30 ± 0.51
B-type natriuretic peptide, pg/mL	1,037 (445-1,862)	—	1,037 (506-1,990)	887 (112-1,433)
HbA_1c_, %	6.30 ± 1.03	—	6.36 ± 1.04	5.97 ± 0.95
Lactate dehydrogenase, units/L	396 (293-614)	—	394 (293-684)	418 (285-527)
Cholesterol, mg/dL	141.2 ± 47.6	187.0 ± 48.2^b^	133.9 ± 45.9	138.8 ± 32.7
LDL, mg/dL	85.7 ± 38.4	112.8 ± 35.3	81.3 ± 39.5	86.2 ± 20.5
HDL, mg/dL	34.4 ± 14.7	60.0 ± 16.8^b^^,^^c^	29.8 ± 9.4	33.2 ± 9.1
Triglycerides, mg/dL	113.8 ± 64.2	77.2 ± 44.2^b^	123.8 ± 69.8	96.0 ± 48.7

Values are n (%), mean ± SD, or median (Q1-Q3) and statistically compared using the chi-squared test or Fisher exact test as appropriate for categorical variables and the 2-group Student’s *t*-tests or Wilcoxon rank-sum tests as appropriate for continuous variables. Assumptions of normality were assessed graphically with histograms and Q-Q plots.

ACE = angiotensin converting enzyme; ARB = angiotensin receptor blocker; HDL = high-density lipoprotein; LDL = low-density lipoprotein; NR = nonresponders; R = responders; VA-ECMO = venoarterial extracorporeal membrane oxygenation; VAD = ventricular assist device.

a NR group vs responder group; *P* < 0.05.

b Control group vs NR group; *P* < 0.05.

c Control group vs R group; *P* < 0.05.

### Circulating targeted lipid profiling

#### Circulating targeted lipid profiling in responders and nonresponders at time of LVAD implantation

**Figure 3:**
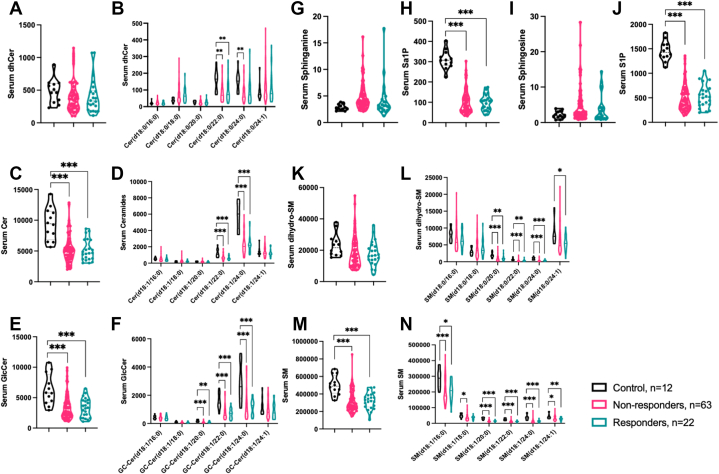
Circulating SLs Pre-LVAD Implantation in NR and R Groups (A and B) Total dhCer levels are consistent across all groups compared to control subjects. DhCer(22:0) and dhCer(24:0) levels are decreased in NR and R groups compared to the control group. (C and D) Total Cer, Cer(22:0). and Cer(24:0) levels are decreased in NR and R groups compared to the control group. (E and F) Total GlcCer, GlcCer (20:0), GlcCer(22:0), and GlcCer(24:0), levels are decreased in all groups compared to the control group. (G and H) Sphinganine levels are consistent in all groups compared to the control group, whereas Sa1P levels are decreased in NR and R groups compared to the control group. (I and J) Sphingosine levels are consistent in all groups compared to control group, whereas S1P levels are decreased in NR and R groups compared to the control group. (K and L) Total dhSM levels are consistent across all groups compared to the control group. DhSM(20:0), dhSM(22:0), and dhSM(24:0) levels are decreased in NR and R groups compared to the control group. DhSM(24:1) levels are lower in R group only compared to control group. (M and N) Total SM, SM(16:0), SM(20:0), SM(22:0), SM(24:0), and SM(24:1) levels are decreased in NR and R groups compared to the control group. SM(18:0) levels are lower in NR group only compared to the control group. All graphs in pmol lipid/mL serum. Mixed-model comparisons of concentrations of sphingolipids measured by liquid chromatography with tandem mass spectrometry for control, NR, and R groups. Multiple comparisons were performed using the Benjamini-Krieger-Yekutieli false discovery rate approach. ∗*P* < 0.05; ∗∗*P* < 0.01; ∗∗∗*P* < 0.001. SL = sphingolipid; other abbreviations as in Figures 1 and 2.

We found that total circulating Cer, GlcCer, and SM levels were lower in both nonresponder and responder groups compared to control group at the time of LVAD implantation (Figure 3), whereas total circulating levels of dhCer, sphinganine, sphingosine, and dhSM were not different across all groups (Figures 3A, 3G, 3I, 3K). These differences were reflected primarily by very long-chain dhCer(22:0, 24:0) (Figure 3B), Cer(22:0, 24:0) (Figures 3C and 3D), and GlcCer(20:0, 22:0, 24:0) (Figures 3E and 3F). Circulating levels of S1P and Sa1P were lower in both nonresponder and responder groups compared to control group (Figures 3H and 3J). Short- and long-chain dhSM(20:0, 22:0, 24:0, and 24:1) (Figure 3L) and SM(16:0, 20:0, 22:0, 24:0, and 24:1) levels were lower in nonresponders and responders compared to control subjects. (Figures 3M and 3N).

#### Circulating targeted lipid profiling in responders and nonresponders at the post-LVAD therapy time point

Of HF patients who underwent LVAD implantation, 47 went on to be explanted or transplanted allowing for longitudinal sample studies between the 2 groups (Figure 2). Baseline demographic, laboratory, and echocardiographic data for this subcohort of patients stratified by nonresponders and responders are presented in Supplemental Table 1. Following LVAD support, nonresponders had significantly higher levels of very long-chain dhCer(22:0, 24:0), Cer(24:0), and dhSM(24:0) levels compared to pre-LVAD implantation levels (Table 2). Total levels of circulating dhCer, Cer, GlcCer, dhSM, and SM were unchanged in nonresponders. However, S1P and Sa1P after LVAD implantation were significantly lower in the responders compared to the nonresponders (Table 2). The rest of the SLs remained overall unchanged in responders after LVAD support (Supplemental Table 2). After correcting for LVAD support duration, we found that longer LVAD duration is associated with higher levels of sphinganine (β = 0.057; 95% CI: 0.011-0.089; *P* < 0.001) and sphingosine (β = 0.15; 95% CI: 0.11-0.19; *P* < 0.001), independent of response status.

**Table 2:** Summary Statistics for LC-MS/MS–Measured Serum SLs in NR and R Groups Pre- and Post-LVAD Support

Targeted Lipid Species	Control Subjects (n = 12)	Nonresponders		Responders	
		Pre-LVAD (n = 63)	Post-LVAD (n = 36)	Pre-LVAD (n = 22)	Post-LVAD (n = 11)
DhCer d18:0/16:0	20.82 ± 9.53	25.4 ± 12.2	24.92 ± 12.73	21.5 ± 9.0	23.37 ± 9.61
DhCer d18:0/18:0	38.59 ± 12.48	71.3 ± 49.8	62.41 ± 47.47	68.2 ± 40.4	76.79 ± 49.35
DhCer d18:0/20:0	27.32 ± 8.13	25.8 ± 13.5	25.76 ± 15.46	25.5 ± 14.0	26.01 ± 12.40
DhCer d18:0/22:0	159.74 ± 62.03	80.3 ± 48.1^a^	122.5 ± 92.4^b^	90.5 ± 63.1^c^	120.0 ± 60.2
DhCer d18:0/24:0	152.27 ± 57.49	73.9 ± 45.7^c^	129.7 ± 112.7^d^	87.4 ± 72.1	136.4 ± 76.5
DhCer d18:0/24:1	89.54 ± 51.52	117.2 ± 84.8	118.6 ± 84.1	114.3 ± 91.8	116.6 ± 66.7
DhCer total	488.3 ± 184.9	393.8 ± 233.0	483.9 ± 340.3	407.4 ± 266.7	499.1 ± 261.2
Cer d18:1/16:0	549.7 ± 152.1	501.0 ± 298.1	502.3 ± 177.4	466.6 ± 199.3	491.1 ± 172.4
Cer d18:1/18:0	194.0 ± 64.4	277.3 ± 207.4	228.1 ± 116.3	252.8 ± 108.4	244.0 ± 101.4
Cer d18:1/20:0	185.2 ± 54.2	179.6 ± 93.0	162.4 ± 63.2	167.8 ± 63.5	153.9 ± 44.8
Cer d18:1/22:0	1,226.2 ± 488.6	693.1 ± 341.1^e^	814.2 ± 348.0^a^	630.5 ± 240.3^e^	832.4 ± 267.6^c^
Cer d18:1/24:0	5,794.1 ± 1,552.4	2,199.3 ± 1,104.1^e^	3,430 ± 1,315^b^^,^^e^	2,279.8 ± 1,028.6^e^	3,527 ± 1,241^e^
Cer d18:1,24:1	1,370.2 ± 505.1	1,354.3 ± 554.6	1,260 ± 442	1,243.1 ± 451.1	1,201 ± 319
Cer total	9,422.0 ± 2,639.8	5,263.2 ± 2,287.6^e^	6,460 ± 2,533^e^	5,100.6 ± 1,730.9^e^	6,520 ± 2,030^e^
GlcCer d18:1/16:0	429.9 ± 127.6	412.2 ± 151.7	408.8 ± 143.9	403.0 ± 155.8	436.1 ± 175.1
GlcCer d18:1/18:0	112.6 ± 45.8	80.1 ± 37.9	83.6 ± 30.0	86.7 ± 49.4	79.9 ± 28.9
GlcCer d18:1/20:0	185.8 ± 74.0	104.8 ± 62.9^e^	122.1 ± 63.2^c^	103.1 ± 54.1^a^	107.7 ± 42.1^c^
GlcCer d18:1/22:0	1,560.3 ± 552.2	738.3 ± 470.1^e^	961.1 ± 552.5^d^	693.5 ± 337.6^e^	841. ± 306.6^a^
GlcCer d18:1/24:0	2,856.2 ± 1,301.9	1,144.8 ± 869.1^e^	1,620.8 ± 950.0^e^	1,154.0 ± 528.0^e^	1,638.6 ± 778.3^a^
GlcCer d18:1/24:1	1,137.1 ± 567.7	918.0 ± 548.4	822.3 ± 419.6	882.5 ± 528.0	845.9 ± 437.1
GlcCer total	6,281.9 ± 2,522.8	3,398.1 ± 2,021.2^e^	4,018.6 ± 2,044.6^a^	3,322.8 ± 1,565.2^e^	3,949.9 ± 1,705.6^c^
DhSM d18:0/16:0	8,027.8 ± 1,942.2	6,706.3 ± 3,296.4	6,180 ± 2,555	6,482.7 ± 2,515.9	6,894 ± 2,862
DhSM d18:0/18:0	2,994.6 ± 1,072.9	3,963.8 ± 2,784.6	2,621.1 ± 1,483.9	4,120.5 ± 2,476.5	4,106.4 ± 2,984.3
DhSM d18:0/20:0	1,979.7 ± 768.1	950.1 ± 774.2^e^	907.2 ± 635.4^a^	984.2 ± 764.0^a^	1,256.7 ± 744.8
DhSM d18:0/22:0	703.9 ± 355.9	321.1 ± 320.6^e^	339.6 ± 227.1^a^	325.2 ± 281.2^a^	466.2 ± 289.2
DhSM d18:0/24:0	944.8 ± 371.9	342.9 ± 305.6^e^	518.8 ± 336.7^b^^,^^e^	357.0 ± 20.7^e^	542.6 ± 262.4^a^
DhSM d18:0/24:1	8,593.3 ± 3,467.0	5,975.0 ± 3,511.6	5,206.7 ± 2,261.5^c^	5,526.3 ± 2,226.2^c^	5,674.7 ± 2,743.0
DhSM total	23,244.3 ± 7,365.8	18,259.2 ± 9,927.8	15,773.5 ± 6,854.9	17,795.8 ± 7,751.1	18,989.9 ± 9,306.3
SM d18:1/16:0	286,698 ± 56,809	199,901.6 ± 72,037.6^e^	206,902.1 ± 73,913.6^c^	205,837.1 ± 58,951.2^c^	220,955.6 ± 69,250.0
SM d18:1/18:0	48,346.8 ± 12,632.5	33,416.1 ± 17,269.7^c^	29,833.4 ± 12,046.6^a^	34,488.2 ± 11,142.2	32,919.4 ± 13,309.2
SM d18:1/20:0	30,182.3 ± 6,848.0	13,549.4 ± 5,619.6^e^	14,841.6 ± 5,649.7^e^	13,962.5 ± 4,836.1^e^	15,907.0 ± 5,781.0^e^
SM d18:1/22:0	25,108.7 ± 6,611.0	11,433.7 ± 5,926.9^e^	13,093.2 ± 5,305.1^e^	11,303.7 ± 4,258.8^e^	14,961.6 ± 4,979.4^e^
SM d18:1/24:0	36,781.8 ± 15,503.4	13,038.6 ± 11,075.4^e^	16,006.6 ± 9,099.1^e^	13,033.5 ± 7,036.5^e^	19,204.1 ± 9,546.0^e^
SM d18:1/24:1	43,185.7 ± 15,823.4	30,059.0 ± 14,170.4^c^	26,162.5 ± 10,166.3^e^	27,722.8 ± 9,497.8^a^	28,181.0 ± 12,524.8^c^
SM total	498,795 ± 111,740	316,862.6 ± 125,511.1^e^	322,738.4 ± 119,083.4^e^	322,014.3 ± 93,560.4^e^	349,378.9 ± 116,039.2^c^
Sphinganine	2.83 ± 0.56	4.85 ± 2.6	3.65 ± 1.76	4.22 ± 3.5	4.48 ± 3.15
Sphingosine	2.18 ± 1.07	4.83 ± 5.1	2.69 ± 3.24	3.88 ± 3.9	2.98 ± 2.92
Sa1P	309.8 ± 46.8	102.7 ± 57.9^e^	131.7 ± 79.3^e^	98.3 ± 38.8^e^	77.7 ± 25.9^e^^,^^f^
S1P	1,425.1 ± 245.1	496.7 ± 273.9^e^	527.9 (324.5-902.2)^e^	525.5 ± 235.1^e^	379.8 (308.4-547.6)^e^^,^^f^

Values are mean ± SD or median (Q1-Q3). Mixed-model comparisons of concentrations of SLs measured by LC-MS/MS for control, NR, and R groups. Multiple comparisons of all SL serum data were performed using the Benjamini-Krieger-Yekutieli false discovery rate approach. Units are pmol lipid/mL serum.

Cer = ceramide; d = dihydroxy; Dh = dihydro; GlcCer = glucosylceramide; LC-MS/MS = liquid chromatography with tandem mass spectrometry; LVAD = left ventricular assist device; S1P = sphingosine-1-phosphate; Sa1P = sphinganine-1-phosphate; SL = sphingolipid; SM = sphingomyelin; other abbreviations as in Table 1.

a *P* < 0.01.

b *P* < 0.05.

c *P* < 0.05.

d *P* < 0.01 vs pre-LVAD of respective group.

e *P* < 0.001 vs donor.

f *P* < 0.05 vs post-NR.

### Myocardial targeted lipid profiling

#### Myocardial targeted lipid profiling in responders and nonresponders at the post-LVAD therapy time point

From our cohort of 99 advanced HF patients, 31 were included in our targeted lipid panel analysis and compared to control donor tissue (Figure 2). Baseline demographic, laboratory, and echocardiographic data are presented in Supplemental Table 3 for control subjects, nonresponders, and responders.

**Figure 4:**
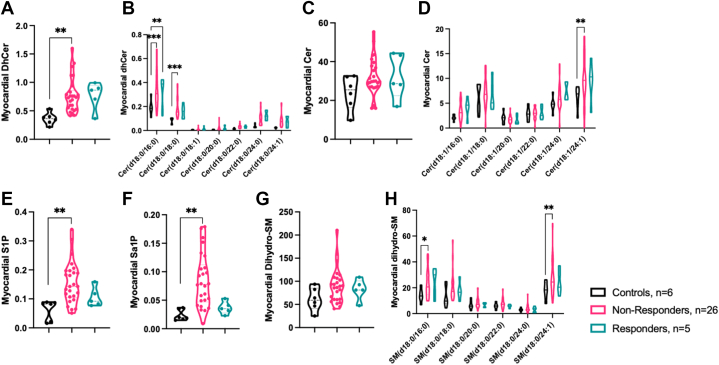
Myocardial SLs in NR and R Groups at Time of LVAD Implantation (A and B) Increased levels of myocardial dhCer in both NR and R groups (*P* = 0.064) compared to the control group. DhCer(16:0) was higher in both NR and R groups compared to the control group, whereas dhCer(18:0) levels were higher in NR group only and dhCer(24:0) was higher in R group only (*P* = 0.062) compared to the control group. (C and D) Consistent levels of myocardial Cer in all groups compared to the control group. Cer(24:1) levels were higher in NR and R groups (*P* = 0.088) compared to the control group. (E) Increased levels of myocardial S1P in NR group compared to the control group. (F) Increased levels of myocardial Sa1P in NR group compared to both control and R groups (*P* = 0.064). (G and H) Consistent levels of myocardial dhSM in all groups compared to the control group. DhSM(16:0) levels were higher in both NR and R groups (*P* = 0.075) compared to the control group, whereas dhSM(24:1) levels were higher in NR group only compared to the control group. (All graphs in pmol lipid/mg tissue. One-way analysis of variance was used to compare concentrations of SLs measured by liquid chromatography with tandem mass spectrometry for control, NR, and R groups. Significant analyses of variance were followed by Sidak-adjusted post hoc pairwise tests. ∗*P* < 0.05; ∗∗*P* < 0.01; ∗∗∗*P* < 0.001). Abbreviations as in Figure 1, Figure 2, Figure 3.

Compared to control subjects, targeted lipid profiling performed in cardiac samples from HF patients at the time of LVAD implantation showed higher levels of total myocardial dhCer, reflecting higher levels of long-chain dhCer(16:0) in both nonresponders and responders, and dhCer(18:0) and very long-chain Cer(24:1) in nonresponders only (Figures 4A to 4D). In addition, compared to control subjects, only nonresponders displayed higher levels of S1P, Sa1P, and dhSM(16:0, 24:1) (Figures 4E to 4H). Interestingly, nonresponders had elevated Sa1P levels compared to responders (Figure 4F).

The rest of the SLs remained grossly unchanged between nonresponders and responders and are included in Supplemental Table 4.

#### Myocardial targeted lipid profiling in responders and nonresponders at the post-LVAD therapy time point

Detailed absolute levels of myocardial SLs from 11 patients at time of myocardial recovery are included in Table 3. After LVAD support, nonresponders and responders had similar levels of total dhCer and Cer, with increased levels of long-chain dhCer(16:0). In addition, responders had increased levels of total dhSM including dhSM(24:1), total SM including SM(24:1), sphingosine (*P* = 0.072), and Sa1P compared to nonresponders (Table 3).

**Table 3:** Means for LC-MS/MS–Measured Cardiac SLs in NR and R Groups After LVAD Support

Targeted Lipid Species	Nonresponders (n = 6)	Responders (n = 5)	*P* Value
DhCer d18:0/16:0	3.74 ± 1.58	2.48 ± 0.53	**0.004**
DhCer d18:0/18:0	3.20 ± 0.37	2.79 ± 0.41	0.81
DhCer d18:0/20:0	0.50 ± 0.26	0.51 ± 0.08	>0.99
DhCer d18:0/22:0	0.89 ± 0.33	0.66 ± 0.11	0.98
DhCer d18:0/24:0	0.91 ± 0.38	0.91 ± 0.29	>0.99
DhCer d18:0/24:1	1.10 ± 0.36	0.99 ± 0.27	0.99
DhCer total	10.36 ± 2.33	8.35 ± 1.32	0.12
Cer d18:1/16:0	50.14 ± 33.18	32.85 ± 10.81	0.44
Cer d18:1/18:0	18.74 ± 4.75	20.08 ± 4.37	>0.99
Cer d18:1/20:0	8.03 ± 3.17	5.30 ± 1.01	>0.99
Cer d18:1/22:0	56.88 ± 22.04	41.23 ± 6.34	0.55
Cer d18:1/24:0	51.30 ± 16.62	53.11 ± 12.33	>0.99
Cer d18:1/24:1	86.34 ± 25.03	100.14 ± 16.13	0.68
Cer total	271.45 ± 83.04	252.72 ± 38.70	0.65
GlcCer d18:1/16:0	5.08 ± 4.02	3.96 ± 1.40	0.99
GlcCer d18:1/18:0	4.52 ± 1.85	5.77 ± 2.59	0.98
GlcCer d18:1/20:0	3.30 ± 1.23	2.97 ± 0.93	>0.99
GlcCer d18:1/22:0	12.45 ± 5.87	14.34 ± 2.44	0.86
GlcCer d18:1/24:0	9.54 ± 3.61	13.19 ± 2.69	0.26
GlcCer d18:1/24:1	7.98 ± 2.06	11.99 ± 2.74	0.17
GlcCer total	42.89 ± 15.08	52.33 ± 6.77	0.23
DhSM d18:0/16:0	153.75 ± 63.24	93.34 ± 24.30	0.82
DhSM d18:0/18:0	68.67 ± 19.56	61.07 ± 11.40	>0.99
DhSM d18:0/20:0	132.83 ± 56.06	145.87 ± 23.29	>0.99
DhSM d18:0/22:0	32.46 ± 9.99	31.65 ± 1.91	>0.99
DhSM d18:0/24:0	54.89 ± 17.35	63.89 ± 7.37	>0.99
DhSM d18:0/24:1	731.55 ± 198.98	1,227.33 ± 199.62	**<0.001**
DhSM total	1,174.17 ± 282.01	1,623.18 ± 193.85	**0.014**
SM d18:1/16:0	2,859.16 ± 592.72	3,070.73 ± 496.62	0.88
SM d18:1/18:0	1,115.62 ± 328.90	1,090.45 ± 204.34	>0.99
SM d18:1/20:0	715.40 ± 263.46	774.74 ± 141.68	0.99
SM d18:1/22:0	924.68 ± 448.43	1,178.32 ± 202.20	0.76
SM d18:1/24:0	731.89 ± 195.43	1,222.31 ± 198.96	0.10
SM d18:1/24:1	1,182.81 ± 179.82	1,959.04 ± 324.85	**0.001**
SM total	7,529.59 ± 1,408.92	9,295.61 ± 808.23	**0.035**
Sphinganine	1.00 ± 0.44	1.20 ± 0.34	0.42
Sphingosine	6.56 ± 1.78	8.97 ± 2.15	0.072
Sa1P	0.22 ± 0.15	0.46 ± 0.16	**0.036**
S1P	0.15 ± 0.09	0.20 ± 0.12	0.46

Values are mean ± SD unless otherwise indicated. **Bold***P* values are statistically significant. One-way ANOVA was used to compare concentrations of SLs measured by LC-MS/MS NR and R groups. Significant ANOVAs were followed by Sidak-adjusted post hoc pairwise tests.

ANOVA = analysis of variance; other abbreviations as in Tables 1 and 2.

#### Correlation analysis between circulating SLs at time of LVAD support and known predictors of myocardial recovery

**Figure 5:**
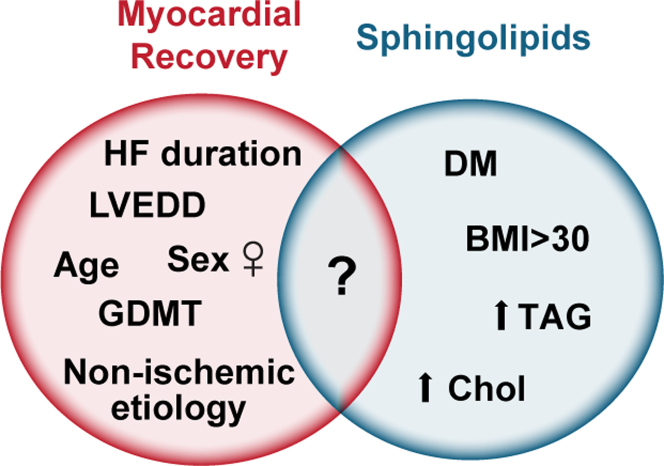
Variables Associated With Myocardial Recovery and SLs Venn diagram shows the variables associated with myocardial recovery and SLs. BMI = body mass index in kg/m^2^; Chol = cholesterol; DM = diabetes mellitus; GDMT = guideline-directed medical therapy; LVEDD = left ventricular end-diastolic diameter; TAG = triacylglycerol; other abbreviations as in Figures 2 and 3.

Previous studies have shown that female patients with nonischemic etiology of HF and, with shorter duration of HF symptoms are more likely to improve significantly their cardiac structure and function post-LVAD support.^4^^,^^5^^,^^8^^,^^46^, ^47^, ^48^ We analyzed the relationship between the circulating pre-LVAD SLs and these clinical factors that are known to facilitate myocardial recovery during LVAD therapy: sex; duration of HF symptoms; HF etiology; and LVEDD. Given the body of evidence associating certain SLs with the metabolic syndrome, diabetes mellitus, and obesity,^49^, ^50^, ^51^, ^52^ we also examined SLs’ association with HbA_1c_ and body mass index in our HF cohort (Figure 5).

Of the 62 SLs measured, 41 were associated with at least 1 of the clinical variables described. We focused on the relationships between SLs with ΔLVEDD and ΔLVEF in nonresponders and responders.

**Figure 6:**
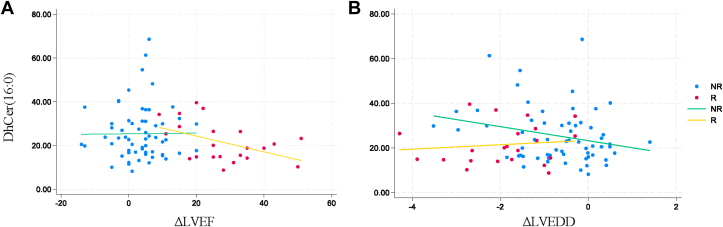
Circulating Levels of Pre-LVAD dhCer(16:0) Associated With LVEF and LVEDD Changes After LVAD Support in NR and R Groups (A) Linear regression showing pre-LVAD serum dhCer(16:0) negatively associated with higher ΔLVEF in R group (linear regression coefficient: β: −0.36; 95% CI: −0.68 to −0.04; *P* = 0.029). No significant associations in NR group. (B) Linear regression showing pre-LVAD serum dhCer(16:0) negatively associated with higher ΔLVEDD in NR group (linear regression coefficient: β: −3.15; 95% CI: −6.29 to −0.020; *P* = 0.049). No significant associations in R group. Abbreviations as in Figure 1, Figure 2, and 5.

We found that in responders, lower circulating dhCer(16:0) was associated with higher ΔLVEF (β: −0.36; 95% CI: −0.68 to −0.04; *P* = 0.029) (Figure 6A). Additionally, lower circulating levels of dhCer(18:0) and dhCer(20:0) were associated with higher ΔLVEF in responders (Supplemental Table 5). In nonresponders, higher circulating dhCer(16:0) was associated with lower ΔLVEDD (β: −3.15; 95% CI: −6.29 to −0.020; *P* = 0.049) (Figure 6B), and with lower GlcCer(20:0), GlcCer(24:1), dhSM(16:0), and PC(36:1). In responders, lower levels of circulating dhSM(20:0), dhSM(22:0), PC(36:0), PC(38:3), PC(38:4), PC(38:6), diacylglycerol species, and total triacylglycerol levels were associated with higher ΔLVEDD (Supplemental Table 5). We did not find any associations among sex, age, HF duration, and SL levels.

#### S1P signaling as a potential target in myocardial recovery

**Figure 7:**
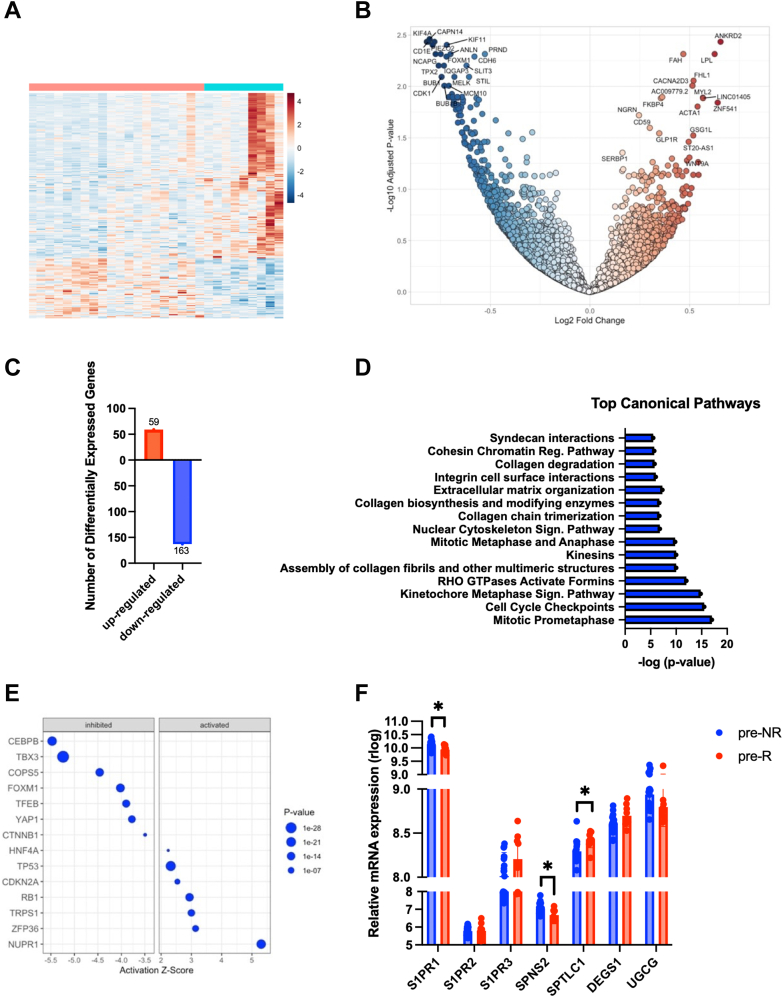
S1P Signaling as a Potential Target to Enhance LVAD-Mediated Myocardial Recovery (A) Heatmaps of 20 pre-NR with high circulating S1P vs 9 pre-R with low circulating S1P. (B) Volcano plots of differentially expressed genes. (C) Number of differentially expressed gene in pre-NR group with high circulating S1P compared to pre-R group with low circulating S1P. (D) Top canonical pathways and biofunctions identified through ingenuity pathway analysis. (E) Dotplot of transcription regulators with highest activation z-scores. CEBPB and TBX3 were described with the highest inhibition z-score, whereas NUPR1 was described with the highest activation z-score in pre-NR group compared to pre-R group. (F) Messenger RNA (mRNA) expression of genes involved in S1P signaling and sodium glucose pathways. S1PR1, SPNS2, and UGCG (*P* = 0.082) are elevated in pre-NR group compared to pre-R group. S1PR3 (*P* = 0.082), SPTLC1 and DEGS1 (*P* = 0.082) are reduced in pre-NR group compared to pre-R group. (Multiple Student’s *t*-tests were followed by Benjamini-Krieger-Yekutieli false discovery rate approach. ∗*P* < 0.05). Abbreviations as in Figures 1 and 2.

To better understand the contribution of SLs in myocardial recovery, and given our findings showing higher levels of myocardial S1P and Sa1P in nonresponders pre-LVAD, we performed quantitative transcriptomics on heart tissues from nonresponders and responders. Hence, we divided our initial cohort to nonresponders with high levels of circulating S1P vs responders with low levels of circulating S1P levels before LVAD implantation. We then performed RNA isolation and sequencing on their respective cardiac tissues collected at time of LVAD implantation. We used a 5% false discovery rate with DeSeq2 software (version 1.42.1) and identified 222 differentially expressed genes between the high S1P nonresponders and the low S1P responders (Figures 7A to 7C). We performed ingenuity pathway analysis as a bioinformatics approach to categorize enriched gene groups among the differentially expressed pathways between the 2 groups. We note that the top canonical pathways that were down-regulated in nonresponders were mitotic prometaphase, cell cycle checkpoints, RHO guanosine triphosphatases activate formins, assembly of collagen fibrils and other multimeric structures, and mitotic metaphase and anaphase (Figure 7D). Among the transcription factors with higher z-score identified were CEBPB and TBX3 that were inhibited and NUPR1 that was activated in high-S1P patients compared to low-S1P patients (Figure 6E). Additionally, RNA-sequencing revealed a reduction in *S1PR1* and *SPNS2* messenger RNA expression in responders compared to nonresponders (Figure 6F). *S1PR3* levels were trending up in responders compared to nonresponders, whereas there were no changes in *S1PR2* levels between the 2 cohorts. From the enzymes involved in the regulation of the de novo and salvage pathways of Cer, the gene expression of 3 key enzymes were significantly different between nonresponders and responders. *SPTLC1* and *DEGS1* expression were increased in responders whereas *UGCG* expression was decreased in responders (Figure 6F).

## Discussion

SLs are a class of bioactive lipids that play crucial role in cell membrane structure and function, signaling, and cellular processes such as apoptosis, inflammation, and differentiation.^16^^,^^17^^,^^32^^,^^33^^,^^53^ Their involvement in cardiovascular diseases has been increasingly studied, because SLs have been linked to various pathophysiological mechanisms including structure and function of cardiac cells, heart cell survival, cardiac remodeling, and inflammatory response.^54^, ^55^, ^56^, ^57^, ^58^ In HF, SL metabolism is altered, with changes in the levels of specific SLs such as Cer. These alterations may contribute to the pathogenesis of HF by promoting inflammation, fibrosis, and apoptosis of cardiomyocytes. However, the role and changes in SL balance in myocardial recovery have not been fully elucidated.

Our findings indicate an accumulation of circulating SLs such as dhCer(22:0), dhCer(24:0), Cer(24:0), and dhSM(24:0) after LVAD support in nonresponders. Prior studies have described elevated levels of Cer in healthy patients to assess cardiovascular risk, in patients with moderate cardiomyopathy or in advanced HF undergoing LVAD placement, with no change after LVAD support.^20^^,^^59^, ^60^, ^61^ However, in our study, we studied a larger cohort of exclusively advanced HF patients with a range of etiologies for which we include phenotyping of the myocardial structural and functional response following LVAD support. As emphasized by the National Heart, Lung, and Blood Institute Working Group on myocardial recovery,^8^ a critical opportunity in the field of cardiac recovery after LVAD is for studies to correlate functional cardiac readouts with molecular or cellular findings by examining tissue from patients with various degrees of LVAD-mediated myocardial functional recovery (ie, responders vs nonresponders). This way, the studies could start shedding light on whether the observed biological changes are associated with cardiac recovery mechanisms or alternatively represent just epiphenomena related to the LVAD therapy.^8^

Elevated Cer levels have been described to induce apoptosis and inhibit cellular growth, which may worsen myocardial dysfunction in HF.^62^^,^^63^ These lipids may also contribute to cardiac fibrosis, a hallmark of HF, by promoting the activation of fibroblasts and collagen deposition.^56^^,^^64^^,^^65^ In mouse models, the accumulation of cardiac Cer through either the increased entry of FAs in cardiomyocytes, the increased Cer production through de novo biosynthesis, or the decreased Cer breakdown have all been directly related to the development of HF with reduced LVEF.^66^, ^67^, ^68^ Our data showed that nonresponders tended to have higher levels of cardiac Cer compared to nonfailing donor hearts, coupled with increased levels of upstream SLs such as dhCer and Sa1P and downstream SLs such as S1P compared to responders. Interestingly, the increased expression of *SPTLC1* and *DEGS1* in responders, which are key enzymes in the de novo pathway, along with the decreased expression of *UGCG*, involved in salvage pathway, suggests a metabolic flexibility in responders that warrants further investigation. Despite being found at lower concentrations in cardiomyocytes, previous studies have shown that significant changes in dhCer, as observed in nonresponders, are indicative of changes in Cer through the de novo pathway^69^ (Figure 1). The marked increase in dhCer(18:0) levels, which is predominantly produced in skeletal muscles by CERS1, also serves as additional validation of the dynamic SL changes happening in the cardiac tissue of nonresponders.^70^ There is a possibility that nonresponders with increased Cer fail to improve their heart function given the Cer-dependent mechanisms described. Future in vitro and in vivo studies in animal models of HF are warranted to further investigate this hypothesis.

The dilation of the LV, and specifically LVEDD, is a crucial clinical variable in myocardial recovery: it has been associated with greater chance of successful LVAD support cessation (either explantation or decommissioning).^5^^,^^47^ Furthermore, after LVAD support cessation, maintaining a normal ventricular size is among the best indicators of sustainable myocardial recovery.^4^ Cer(16:0) and SM(16:0), downstream by-products of dhCer(16:0), have been described to be positively associated with a higher risk of incident HF and to be elevated in chronic HF patients who died from cardiovascular causes.^59^, ^60^, ^61^ In cardiomyocytes, dhCer(16:0) and Cer(16:0) are produced by CERS5.^69^ In vitro studies have shown an association between Cer synthesis via CERS5 and cardiac hypertrophy and autophagy.^70^, ^71^, ^72^ Interestingly, our findings suggest an association between higher levels of dhCer(16:0) and lower ΔLVEDD in nonresponders, as well as an association between lower levels of dhCer(16:0) and increased ΔLVEF in responders. In addition, responders had lower levels of cardiac dhCer(16:0) after LVAD support than nonresponders. Whether long-chain dhCer(16:0) and its downstream products are related to myocardial recovery and what the role of CERS5 in this process is, merit further investigation.

In addition, our circulating and cardiac lipidomics data showed that nonresponders displayed increased levels of S1P and Sa1P at the time of LVAD implantation. Interestingly, these findings were attenuated in responders at the same time point. In the heart, S1P signaling has been shown to exhibit a cardioprotective effect in pathological cardiac remodeling, and to reduce ischemia size after ischemia/reperfusion injury in mice and porcine models.^56^^,^^73^^,^^74^ Circulating S1P then binds to S1PR1 and S1PR3, which are the predominant S1P receptors within the cardiac milieu.^75^ In the context of HF, human studies have shown that plasma S1P levels were negatively associated with LVEF and NYHA functional class.^76^ Murine studies showed that S1PR1 mediates the negative inotropic effects of S1P in the heart,^55^ whereas selective activation of S1PR3 mediated cardioprotection after ischemia/reperfusion injury.^77^ Given our findings, it is plausible that low S1P levels may be a biomarker associated with myocardial recovery and its downstream mechanism of action merits further exploration. Higher levels of cardiac S1P combined with higher tissue expression of S1PR1 in nonresponders and higher S1PR3 in responders may contribute to the enhanced negative inotropic effect and may be a marker of lack of myocardial improvement. Future mechanistic study to explore the cellular and mechanistic effect of S1P and its receptors in human induced-pluripotent stem cell–derived cardiomyocyte model would provide valuable insight.

Finally, the mechanisms through which SLs may affect myocardial recovery have not been established. It is possible that the parallel increases we observed in the levels of dhCer, Cer, and SM reflect a single mechanistic pathway resulting in increased S1P levels that act in both autocrine and paracrine manner. Coordination between several cell types of the divergent actions of S1P toward myocardial recovery merits further exploration. Control of a differential S1PR expression in cardiomyocytes could be a potential therapy to optimize myocardial repair and promote myocardial recovery. With our tissue transcriptomic analysis, we identified CEBPB, TBX3, and NUPR1 as potential targets downstream of S1PR activation through elevated levels of circulating S1P in nonresponders. CEBPB is a transcription factor, participating in a variety of biological processes including cell proliferation, differentiation, and development. Studies in mice and zebrafish have shown that CEBPB represses cardiomyocyte growth and proliferation in the adult mammalian heart and that reduction in CEBPB is a central signal in physiologic hypertrophy and proliferation.^78^ In addition, TBX3 is a transcription factor important in the development and function of the heart’s conduction system. Within the heart TBX3 expression is required for normal size and function of the sinoatrial and atrioventricular nodes. Two recent studies suggested that SLs are associated with incident atrial fibrillation^79^ and sudden cardiac death.^80^ Finally, NUPR1 is a stress-inducible protein that has been described in stress processes and apoptosis in the cancer field^81^ and in cardiomyocyte hypertrophy and cardiac fibrosis induction.^82^^,^^83^ It is plausible that SLs, whose main biological role is to contribute to the integrity of the membrane lipid rafts, may affect assembly microdomains and interact with downstream cascades involving CEBPB, TBX3, and NUPR1; however, future studies will investigate these hypotheses.

### Study limitations

Our study was observational in nature and we cannot infer causation between SL levels and clinical outcomes. Whereas we observed significant associations, future mechanistic studies such as isotope flux tracing studies and in vivo in small animal models and in vitro will be necessary to determine whether SLs influence myocardial recovery and establish causation vs these changes being epiphenomena. In this study, we only had paired serum and myocardial tissue samples for a subset of patients. This limited overlap reduces the power to draw definitive conclusions about the relationship between circulating and myocardial SL profiles and highlights the need for a larger sample size to strengthen these associations. Lastly, our patient population was studied before sodium glucose cotransporter-2 inhibitors were widely used in the management of HF. As such, our data set does not include information on sodium glucose cotransporter-2 inhibitor use and the potential interaction between these agents and SL metabolism. Future basic and clinical studies incorporating contemporary HF therapies and larger patient populations will be required to validate and extend our findings.

## Conclusions

This study indicates the importance of circulating and tissue SLs in HF and myocardial recovery and suggests that enzymes within the SL pathway may serve as potential therapeutic targets. By using serial serum and cardiac tissue samples from a relatively large and well-characterized cohort of advanced HF patients who demonstrated significant structural and functional myocardial improvement, we show different SL profiles associated with reverse remodeling, offering valuable insights into potential metabolic signatures.

Perspectives **COMPETENCY IN MEDICAL KNOWLEDGE:** With the limited availability of donor hearts for transplantation, it is critical to identify mechanisms that promote myocardial recovery and preserve native heart function. This study provides new insight into the role of SLs, particularly the reduction in circulating S1P levels and downstream signaling, in myocardial recovery following LVAD support. Whereas Cer have previously been implicated in the prediction of incident HF,^64^, ^65^, ^66^ this study highlights the potential contribution of Cer and S1P play to the process of myocardial recovery.

**TRANSLATIONAL OUTLOOK:** Emerging studies on SLs in advanced HF and myocardial recovery offer novel mechanistic insights with significant implications for translational medicine, pointing toward new avenues for therapeutic targeting and investigation. This study aligns with previous findings demonstrating that selective activation of S1PR was cardioprotective in models of ischemia-reperfusion injury^73^ and underscores the potential for future mechanistic and preclinical studies to further explore this pathway.

## Funding Support and Author Disclosures

This study was funded by the National Institutes of Health award NHLBI 2T32HL007576-36 (to Dr Kyriakopoulos), NHLBI R01HL135121 (to S.G.D.), NHLBI R01HL132067 (to Dr Drakos), NHLBI R01HL166513 (to S.G.D.), and 1K08HL168315 (to Dr Tseliou). Dr Summers has served as a consultant to and is a shareholder of Centaurus Therapeutics. Dr Drakos has served as a consultant to Abbott. The contents of this manuscript are solely the responsibility of the authors and do not necessarily represent the official views of the National Institutes of Health. All other authors have reported that they have no relationships relevant to the contents of this paper to disclose.
