# Supplementary material for: The Role of Sphingolipids in Myocardial Recovery Mediated by Mechanical Unloading and Circulatory Support
Source: JACC Basic Transl Sci. 2025 Dec 19;11(1):101435. doi: 10.1016/j.jacbts.2025.101435 (PMC12775851; doi:10.1016/j.jacbts.2025.101435)
Supplement: Supplementary Tables 1-5 [file mmc1.docx]

**The Role of Sphingolipids in Myocardial Recovery Mediated by Mechanical Unloading and Circulatory Support**

**Supplementary Appendix**

**Supplementary Table 1.** Baseline demographics and laboratory characteristics in healthy patients, non-responders and responders included in serum lipidomics analysis.
**Supplementary Table 2.** Summary statistics (mean ± standard deviation or median [25^th^-75^th^ percentiles], as appropriate) for LC-MS/MS measured serum SLs in control groups, non-responders and responders at both pre-LVAD and after LVAD support timepoints.
**Supplementary Table 3**. Summary statistics (mean ± SD or median (25^th^-75^th^ percentiles), as appropriate) of baseline demographics and laboratory characteristics in controls, non-responders and responders included in cardiac tissue lipidomics analysis.
**Supplementary Table 4.** Summary statistics (mean ± standard deviation) for LC-MS/MS measured cardiac SLs in control groups, non-responders and responders pre-LVAD.
**Supplementary Table 5.** Linear regressions of pre-LVAD circulating SLs with ΔLVEDD and ΔLVEF in non-responders and responders

**Supplementary Table 1. Baseline demographics and laboratory characteristics in healthy patients, non-responders and responders included in serum lipidomics analysis.**Data are presented using count (%), mean ± standard deviation or median [25th-75th percentiles] and statistically compared using the chi-squared test or Fisher’s exact test as appropriate for categorical variables and the two-group Student’s t-tests or Wilcoxon rank-sum tests as appropriate for continuous variables. Assumptions of normality were assessed graphically with histograms and Q-Q plots.^a^Controls v. Non-responder pre-LVAD, p-value<0.05; ^b^Controls v Non-responder post-LVAD, p-value<0.05; ^c^Controls v. Responder pre-LVAD, p-value<0.05; ^d^Controls v. Responder post-LVAD, p-value<0.0; ^e^ Non-responder pre-LVAD v. Non-responder post-LVAD, p-value<0.05; ^f^ Non-responder pre-LVAD v. Responder pre-LVAD, p-value<0.05; ^g^Non-responder post-LVAD v. Responder post-LVAD, p-value<0.05; ^h^Responder pre-LVAD v. Responder post-LVAD, p-value<0.05.

|  | **Controls (n=12)** | **Non-Responders (n=98)** | | **Responders (n=34)** | |
| --- | --- | --- | --- | --- | --- |
|  |  | **Pre-LVAD (n=63)** | **Post-LVAD (n=35)** | **Pre-LVAD (n=22)** | **Post-LVAD (n=12)** |
| **Age, years** | 53.3 ± 12.9^b^ | 56.6 ± 14.0^e^ | 53.1 ± 10^g^ | 55.1 ± 18.0 | 46.9 ± 15.9 |
| **Male sex, n (%)** | 8 (66.7%)^a^ | 58 (92.1%)^f^ | 32 (91.4%) | 15 (68.2%) | 8 (66.7%) |
| Race |  |  |  |  |  |
| **Caucasian, n (%)** | 12 (100%)^4^ | 55 (91.7%)^f^ | 31 (88.6%) | 10 (71.4%) | 7 (63.6%) |
| Ethnicity |  |  |  |  |  |
| Hispanic, n (%) | 0 (0%) | 3 (5.0%) | 2 (7.4%) | 2 (14.3%) | 2 (18.2%) |
| Body mass index, Kg/m2 | 26.6 ± 4.1 | 29.0 ± 6.2 | 28.9 ± 6.7 | 28.5 ± 5.0 | 29.9 ± 4.2 |
| Medical history |  |  |  |  |  |
| Hx of hypertension, n (%) | --- | 30 (47.6%) | 14 (40.0%) | 13 (59.1%) | 7 (58.3%) |
| Diabetes mellitus, n (%) | --- | 23 (37.1%) | 11 (31.4%) | 6 (27.3%) | 4 (33.3%) |
| Atrial fibrillation, n (%) | --- | 26 (41.3%) | 13 (37.1%) | 7 (33.3%) | 2 (18.2%) |
| Previous thoracotomy, n (%) | --- | 22 (34.9%) | 9 (25.7%) | 3 (13.6%) | 1 (8.3%) |
| **Hx of smoking, n (%)** | 0 (0%)^b,d^ | 26 (41.3%) | 15 (42.9%) | 10 (45.5%) | 6 (50.0%) |
| Hx of ethanol use, n (%) | --- | 23 (38.3%) | 14 (51.9%) | 7 (46.7%) | 6 (50.0%) |
| Hx of substance abuse, n (%) | 0 (0%) | 4 (6.4%) | 4 (11.4%) | 4 (18.2%) | 3 (25.0%) |
| Pre-operative supportive therapies |  |  |  |  |  |
| Inotrope dependency, n (%) | --- | 40 (63.5%) | 5 (27.8%) | 13 (59.1%) | 7 (24.1%) |
| Intra-aortic balloon pump, n (%) | --- | 7 (11.7%) | 8 (22.9%) | 0 (0%) | 0 (0%) |
| Impella/VA-ECMO, n (%) | --- | 2 (3.2%) | 1 (2.9%) | 2 (9.1%) | 1 (8.3%) |
| New York Heart Association class IV, n (%) | --- | 44 (73.3%) | 27 (77.1%) | 11 (73.3%) | 9 (75.0%) |
| **Heart failure duration, months [IQR]** | --- | 74 [48-126]^f^ | 72 [48-144]^g^ | 10 [1-31] | 16 [3.5-39.5] |
| Heart failure etiology | --- |  |  |  |  |
| Ischemic cardiomyopathy, n (%) | --- | 25 (39.7%) | 16 (45.7%) | 7 (31.8%) | 4 (33.3%) |
| Heart failure medications |  |  |  |  |  |
| Beta blocker, n (%) | --- | 43 (68.3%) | 24 (68.6%) | 18 (81.8%) | 10 (83.3%) |
| ARB/ACE inhibitor, n (%) | --- | 36 (57.1%) | 17 (63.0%) | 14 (63.6%) | 9 (75.0%) |
| **Aldosterone antagonist, n (%)** | --- | 40 (63.5%) | 27 (77.1%)^g^ | 10 (45.5%) | 5 (41.7%) |
| **Diuretic, n (%)** | --- | 62 (98.4%) | 34 (97.1%) | 20 (90.9%) | 10 (83.3%) |
| Lipid-lowering medications |  |  |  |  |  |
| **Statins, n (%)** | --- | 31 (49.2%)^f^ | 15 (55.6%)^g^ | 2 (9.1%) | 2 (16.7%) |
| Echocardiographic measurements |  |  |  |  |  |
| Left ventricular ejection fraction, % | --- | 18.7 ± 6.8 | 18.4 ± 7.7 | 20.4 ± 8.8 | 19.5 ± 10.1 |
| Left ventricular end-diastolic diameter, cm | --- | 6.91 ± 1.16 | 6.88 ± 1.12 | 6.42 ± 0.96 | 6.37 ± 1.23 |
| Left ventricular end-systolic diameter, cm | --- | 6.32 ± 1.23 | 6.28 ± 1.08 | 5.79 ± 1.16 | 5.71 ± 1.31 |
| Laboratory measurements |  |  |  |  |  |
| **White blood cell count, ×10^3^/µL** | 5.23 ± 0.87 ^a,b^ | 9.2 ± 4.0 | 9.02 ± 2.44 | 8.7 ± 2.9 | 8.2 ± 2.7 |
| Hemoglobin, g/dL | 14.5 ± 1.0 | 12.5 ± 2.1 | 12.8 ± 2.4 | 12.2 ± 2.0 | 12.1 ± 1.5 |
| Platelet count, ×10^3^/µL | 266.3 ± 55.6 | 199.0 ± 77.4 | 196.6 ± 87.7 | 233.7 ± 79.1 | 232.1 ± 71.0 |
| **Sodium, mEq/L** | 141.3 ± 3.7^a,b,c,d^ | 133.1 ± 5.1 | 133.7 ± 5.1 | 134.5 ± 5.3 | 133.8 ± 5.7 |
| Potassium, mEq/L | 4.38 ± 0.25 | 4.04 ± 0.50 | 4.06 ± 0.45 | 4.03 ± 0.29 | 4.03 ± 0.28 |
| Blood urea nitrogen, mg/dL | --- | 29.4 ± 11.7 | 27.8 ± 10.6 | 25.7 ± 17.3 | 27.3 ± 18.8 |
| Creatinine, mg/dL | 0.90 ± 0.12 | 1.32 ± 0.44 | 1.24 ± 0.36 | 1.45 ± 0.87 | 1.56 ± 0.93 |
| **Blood glucose, g/dL** | 82.7 ± 7.2^a,b^ | 124.1 ± 40.3 | 125.4 ± 36.7 | 107.1 ± 23.2 | 106.7 ± 24.1 |
| Uric acid, mg/dL | --- | 8.4 ± 2.4 | 7.9 ± 2.6 | 7.1 ± 2.6 | 7.7 ± 2.4 |
| **Aspartate aminotransferase, mg/dL** | 22.5 [22.0-23.0]^a,b,c^ | 32 [23-44] | 31 [25-35] | 35 [24-49] | 36 [23-46] |
| Alanine aminotransferase, mg/dL | 18 [12-32] | 27 [22-51] | 30 [24-56] | 32 [18-62] | 31 [17-57] |
| **Alkaline phosphatase, mg/dL** | 62.5 [58.0-68.0]^a,b,c,d^ | 93 [71-119] | 94 [77-123] | 106 [85-128] | 111 [86-136] |
| Total serum protein, g/dL | 7.07 ± 0.23 | 6.95 ± 0.84 | 7.02 ± 0.75 | 7.27 ± 0.88 | 7.30 ± 0.92 |
| **Albumin, g/dL** | 4.58 ± 0.28^a,b,c,d^ | 3.81 ± 0.52 | 3.94 ± 0.44=1 | 3.85 ± 0.42 | 3.89 ± 0.43 |
| Total bilirubin, mg/dL | --- | 1.2 [0.8-1.8] | 1.1 [0.8-1.8] | 1.0 [0.7-1.4] | 1.0 [0.7-1.3] |
| Direct bilirubin, mg/dL | --- | 0.8 [0.6-1.1] | 0.7 [0.2-1.1] | 0.3 [0.2-0.5] | 0.4 [0.2-0.5] |
| International Normalized Ratio (INR) | 0.97 ± 0.05 | 1.33 ± 0.36 | 1.27 ± 0.34 | 1.30 ± 0.51 | 1.20 ± 0.28 |
| B-type natriuretic peptide, pg/mL | --- | 897 [506-1990] | 776 [403-1990] | 887 [112-1433] | 887 [77-1407] |
| Hba1c, % | --- | 6.36 ± 1.04 | 6.24 ± 1.08 | 5.97 ± 0.95 | 6.13 ± 1.02 |
| Lactate dehydrogenase, units/L | --- | 394 [293-684] | 306 [239-558] | 418 [285-527] | 3336 [251-500] |
| **Cholesterol, mg/dL** | 187.0 ± 48.2^a^ | 133.9 ± 45.9 | 156.2 ± 59.3 | 138.8 ± 32.7 | 138.8 ± 32.7 |
| LDL, mg/dL | 112.8 ± 35.3 | 81.3 ± 39.5 | 93.4 ± 48.0 | 86.2 ± 20.5 | 86.2 ± 20.5 |
| **HDL, mg/dL** | 60.0 ± 16.8^a,b,c,d^ | 29.8 ± 9.4 | 27.6 ± 10.3 | 33.2 ± 9.1 | 33.2 ± 9.1 |
| **Triglycerides, mg/dL** | 77.2 ± 44.2^a^ | 123.8 ± 69.8 | 135.6 ± 82.1 | 96.0 ± 48.7 | 96.0 ± 48.7 |

**Supplementary Table 2. Summary statistics (mean ± standard deviation) for LC-MS/MS measured serum sphingolipids in control groups, non-responders and responders at both pre-LVAD and after LVAD support timepoints.**Mixed model comparisons of concentrations of sphingolipids measured by LC-MS/MS for controls, non-responders and responders. Multiple comparisons of all SL serum data were performed using the Benjamini-Krieger-Yekutieli false discovery rate (FDR) approach. Units are pmol lipid/mL serum. (^*^p-value<0.05, **p-value<0.01, ***p-value<0.001 versus donor; ^#^p-value<0.05 versus pre-LVAD of respective group). DAG, diacylglycerol; PC, phosphatidylcholine; TAG, triacylglycerol. Units are pmol lipid/mL serum.

|  |  | **Non-Responders** | | **Responders** | |
| --- | --- | --- | --- | --- | --- |
| Lipid | **Controls (n=12)** | **Pre-LVAD (n=63)** | **Post-LVAD (n=36)** | **Pre-LVAD (n=22)** | **Post-LVAD (n=11)** |
| *PC34:1* | 170820 ± 34253 | 132170.7 ± 38800.2 | 138639 ± 49273 | 144517.0 ± 45702.3 | 148423 ± 588.36 |
| *PC34:2* | 250420 ± 42652 | 180031.7 ± 51264.8^***^ | 195640 ± 59568^*^ | 188985.2 ± 56085.2^*^ | 208261 ± 69230 |
| *PC36:0* | 1868.7 ± 520.4 | 736.7 ± 390.8^***^ | 1044.6 ± 569.9^***,#^ | 843.5 ± 521.4^***^ | 1103.6 ± 478.8^***^ |
| *PC36:1* | 62750.8 ± 19102.6 | 42539.8 ± 23170.2 | 50562.3 ± 29858.8 | 43634.5 ± 20946.4 | 53550.4 ± 31877.5 |
| *PC36:2* | 199759 ± 46382 | 130228.9 ± 47177.9^***^ | 147468 ± 55921^*^ | 130955.7 ± 47746.5^**^ | 156975 ± 66502 |
| *PC36:3* | 139424 ± 34983 | 89534.9 ± 38225.8^**^ | 104824 ± 44863 | 100433.9 ± 46025.9 | 114135 ± 51775 |
| *PC36:4* | 121134 ± 24723 | 90353.2 ± 28256.2^*^ | 92340 ± 32142^*^ | 97111.3 ± 30104.7 | 110387 ± 37057 |
| *PC38:3* | 43435.9 ± 16575.1 | 28900.2 ± 18966.8 | 35216.2 ± 20737.1 | 32992.1 ± 26345 | 41561.7 ± 25302.2 |
| *PC38:4* | 52855.4 ± 13984.8 | 42443.1 ± 16680.5 | 44502.0 ± 17596.5 | 46722.7 ± 22320.5 | 54864.3 ± 22471.7 |
| *PC38:6* | 88039.0 ± 24047.7 | 52222.7 ± 25803.2^***^ | 50114.9 ± 23598.2^***^ | 59013.7 ± 24900.1^*^ | 63744.6 ± 35849.1 |
| *PC40:6* | 38984.9 ± 14450.9 | 25647.3 ± 14924.8 | 23267.2 ± 14115.6^*^ | 27458.1 ± 13707.6 | 32221.5 ± 23855.8 |
| *PC total* | 1169491 ± 242669 | 814809.1 ± 278033.5^**^ | 883618 ± 323911 | 872667.7 ± 307729.2 | 985228 ± 386296 |
| *TAG total* | 1296176 ± 1053700 | 1076025.5 ± 881233.9 | 1224409 ± 842122 | 1016151.6 ± 958529.6 | 1564912 ± 1534834 |
| *DAG16:0/16:0* | 584.7 ± 602.1 | 870.24 ± 593.7 | 977 ± 620 | 713.5 ± 421.9 | 1450.4 ± 1491.9^*,#^ |
| *DAG16:0/16:1* | 261.5 ± 247.8 | 334.5 ± 279.6 | 412 ± 303 | 352.2 ± 259.1 | 603 ± 628 |
| *DAG16:0/18:1* | 4487.2 ± 3767.1 | 5413.1 ± 4587.8 | 5990 ± 3786 | 4693.1 ± 3292.5 | 8276 ± 8391 |
| *DAG16:0/18:2* | 4533.5 ± 3467.4 | 5083.2 ± 3798.6 | 5949.6 ± 4042.8 | 4281.6 ± 3989.4 | 7014.9 ± 5880.7 |
| *DAG16:0/20:4* | 332.0 ± 256.8 | 570.6 ± 333.2 | 498.3 ± 239.9 | 569.7 ± 320.4 | 812.3 ± 633.1^**^ |
| *DAG16:0/22:6* | 250.8 ± 152.3 | 241.3 ± 226.8 | 179.1 ± 150.3 | 198.1 ± 168.3 | 274.7 ± 246.6 |
| *DAG18:1/18:1* | 16090 ± 9592 | 15949.7 ± 12844.3 | 15950 ± 12844 | 13736 ± 11961 | 21008 ± 20644 |
| *DAG18:1/18:2* | 10885 ± 6791 | 10566.7 ± 7238.3 | 12381 ± 8493 | 10002.4 ± 10016.0 | 13944 ± 10949 |
| *DAG18:2/18:2* | 3460.1 ± 2175.0 | 3177.4 ± 1867.5 | 3645.8 ± 2321.0 | 3439.6 ± 3298.1 | 3912.2 ± 2217.4 |
| *DAG total* | 46042.2 ± 30870.8 | 42116.7 ± 30468.1 | 471416± 30539 | 37322.7 ± 32505.7 | 56893 ± 50106 |

**Supplementary Table 3. Baseline demographics and laboratory characteristics controls, non-responders and responders included in cardiac tissue cohort.**Data are presented using count (%) or mean ± standard deviation and statistically compared using the chi-squared test or Fisher’s exact test as appropriate for categorical variables and the two-group Student’s t-tests or Wilcoxon rank-sum tests as appropriate for continuous variables. Assumptions of normality were assessed graphically with histograms and Q-Q plots.^a^Controls v. Non-responder p-value<0.05; ^b^Controls v. Responder p-value<0.05; ^c^Non-responder v. Responder, p<0.05; ^d^Controls v. Non-responder, p<0.0; ^e^Controls v. Responder, p<0.0, ^f^Non-responder v. Responder, p<0.0, ^g^Controls v. Non-responder, p<0.001.

| Variables | Total (n=37) | Controls (n=6) | Non-Responder (n=26) | Responder (n=5) | p-value |
| --- | --- | --- | --- | --- | --- |
| Age, years | 55.3 ± 14.5 | 42.8 ± 6.7 | 58.3 ± 13.6 | 54.8 ± 19.8 | 0.059 |
| Male sex, n (%) | 30 (81.1%) | 3 (30.0%) | 24 (92.3%) | 3 (60.0%) | 0.021 |
| Race |  | | | | |
| Caucasian, n (%) | 34 (91.9%) | 6 (100%) | 23 (88.5%) | 5 (100%) | >0.99 |
| Ethnicity |  |  |  |  |  |
| Hispanic or Latino, n (%) | 2 (5.4%) | 0 (0%) | 2 (7.7%) | 0 (0%) | >0.99 |
| **Body mass index, Kg/m2** | 28.0 ± 4.6 | 24.4 ± 4.2^a^ | 29.3 ± 4.1 | 25.2 ± 4.5 | 0.027 |
| Medical history |  | | | | |
| Hx of hypertension, n (%) | 10 (27.0%) | 0 (0%) | 8 (30.8%) | 2 (40.0%) | 0.32 |
| Diabetes mellitus, n (%) | 11 (30.6%) | 0 (0%) | 9 (36.0%) | 2 (40.0%) | 0.227 |
| Hx of smoking, n (%) | 9 (24.3%) | 0 (0%) | 7 (26.9%) | 2 (40.0%) | 0.30 |
| Hx of ethanol use, n (%) | 7 (18.9%) | 0 (0%) | 6 (23.1%) | 1 (20.0%) | 0.67 |
| Hx of substance abuse, n (%) | 3 (8.1%) | 1 (16.7%) | 1 (3.9%) | 1 (20.0%) | 0.21 |
| **Systolic Blood Pressure, mmHg** | 107.5 ± 16.3 | 125.3 ± 14.8^d^ | 102.7 ± 14.5 | 109.5 ± 11.8 | 0.006 |
| Diastolic Blood Pressure, mmHg | 67.6 ± 11.9 | 69.7 ± 12.6 | 67.3 ± 12.3 | 67.0 ± 10.6 | 0.91 |
| **Cardiac Index** | 2.22 ± 1.04 | 4.05 ± 0.58^g,h^ | 1.70 ± 0.45 | 2.10 ± 0.59 | <0.001 |
| Laboratory Measurements |  | | | | |
| **Sodium, mEq/L** | 138.3 ± 9.2 | 156.0 ± 5.2^g,h^ | 134.3 ± 5.0 | 137.6 ± 1.9 | <0.001 |
| Potassium, mEq/L | 4.04 ± 0.49 | 4.03 ± 0.27 | 4.05 ± 0.56 | 3.94 ± 0.24 | 0.90 |
| Blood urea nitrogen, mg/dL | 26.7 ± 13.3 | 13.8 ± 5.8 | 31.6 ± 12.3 | 16.8 ± 8.3 | 0.001 |
| **Creatinine, mg/dL** | 1.26 ± 0.45 | 0.98 ± 0.24 | 1.39 ± 0.46^c^ | 0.88 ± 0.20 | 0.015 |

**Supplementary Table 4. Means and standard deviation for LC-MS/MS measured cardiac sphingolipids in control groups, non-responders and responders.**One-way ANOVA was used to compare concentrations of sphingolipids measured by LC-MS/MS for controls, non-responders and responders. Significant ANOVAs were followed by Sidak-adjusted post-hoc pairwise tests. Units are pmol lipid/mL serum. Cer, ceramide; GlcCer, glucosylceramide; SM, sphingomyelin; PC, phosphatidylcholine; ; TAG, triacylglycerol, DAG, diacylglycerol;.

| Lipid | Controls (n=6) | Non-Responders (n=26) | Responders (n=5) |
| --- | --- | --- | --- |
| *Cer total* | 24.0 ± 8.8 | 32.19 ± 9.54 | 32.24 ± 11.56 |
| *GlcCer d18:1/16:0* | 0.30 ± 0.13 | 0.54 ± 0.42 | 0.66 ± 0.49 |
| *GlcCer d18:1/18:0* | 0.58 ± 0.26 | 0.81 ± 0.33 | 0.73 ± 0.29 |
| *GlcCer d18:1/20:0* | 1.49 ± 0.85 | 1.45 ± 0.90 | 1.31 ± 0.66 |
| *GlcCer d18:1/22:0* | 5.12 ± 1.99 | 5.82 ± 4.24 | 5.03 ± 3.24 |
| *GlcCer d18:1/24:0* | 4.4 ± 2.02 | 6.94 ± 6.21 | 6.87 ± 6.1 |
| *GlcCer d18:1/24:1* | 4.3 ± 2.3 | 5.85 ± 5.45 | 5.88 ± 5.69 |
| *GlcCer total* | 16.2 ± 7.1 | 21.42 ± 16.68 | 20.48 ± 15.25 |
| *SM d18:1/16:0* | 522.8 ± 212.6 | 528.3 ± 201.4 | 539.2 ± 159.8 |
| *SM d18:1/18:0* | 143.7 ± 63.9 | 179.6 ± 62.5 | 143.0 ± 44.9 |
| *SM d18:1/20:0* | 56.7 ± 26.1 | 51.9 ± 15.5 | 52.8 ± 20.5 |
| *SM d18:1/22:0* | 172.0 ± 70.7 | 156.5 ± 54.2 | 141.8 ± 46.5 |
| *SM d18:1/24:0* | 127.3 ± 51.0 | 136.5 ± 83.2 | 95.4 ± 44.2 |
| *SM d18:1/24:1* | 185.8 ± 75.5 | 269.5 ± 113.6 | 242.3 ± 85.9 |
| *SM total* | 1235.2 ± 479.3 | 1353.3 ± 493.0 | 1244.2 ± 309.5 |
| *PC34:1* | 1998.8 ± 687.6 | 2039.5 ± 1028.9 | 2198.8 ± 590.0 |
| *PC34:2* | 2125.5 ± 744.8 | 1902.8 ± 936.8 | 2050.8 ± 579.8 |
| *PC36:0* | 41.1 ± 19.8 | 35.6 ± 26.4 | 30.4 ± 13.8 |
| *PC36:1* | 677.0 ± 280.4 | 722.4 ± 483.9 | 630.0 ± 221.1 |
| *PC36:2* | 1000.2 ± 356.7 | 902.2 ± 490.8 | 914.0 ± 232.0 |
| *PC36:3* | 387.1 ± 143.5 | 387.7 ± 172.3 | 391.4 ± 80.8 |
| *PC36:4* | 1340.3 ± 496.4 | 1175.2 ± 794.6 | 1315.5 ± 300.8 |
| *PC total* | 7570.0 ± 2589.2 | 7165.4 ± 3830.1 | 7530.9 ± 1813.2 |
| *Sphinganine* | 0.062 ± 0.039 | 0.076 ± 0.054 | 0.073 ± 0.037 |
| *Sphingosine* | 0.381 ± 0.125 | 0.538 ± 0.444 | 0.591 ± 0.267 |
| *TAG total* | 61486.1 ± 45391.6 | 102859.5 ± 120735.1 | 148851.5 ± 166983.2 |
| *DAG 16:0/16:0* | 21.4 ± 18.8 | 29.6 ± 34.7 | 31.6 ± 25.8 |
| *DAG 16:0/18:1* | 165.8 ± 111.2 | 226.9 ± 247.8 | 231.2 ± 165.8 |
| *DAG 16:0/18:2* | 138.4 ± 69.4 | 241.4 ± 319.8 | 274.8 ± 281.2 |
| *DAG 18:1/18:1* | 377.8 ± 237.5 | 334.9 ± 275.3 | 369.3 ± 198.2 |
| *DAG 18:1/18:2* | 244.3 ± 165.9 | 213.8 ± 172.3 | 255.6 ± 168.0 |
| *DAG 18:2/18:2* | 19.1 ± 13.7 | 26.3 ± 30.2 | 41.3 ± 46.3 |
| *DAG total* | 966.8 ± 479.1 | 1072.9 ± 1028.8 | 1203.7 ± 840.8 |

**Supplementary Table 5: Linear regressions of pre-LVAD circulating sphingolipids with ΔLVEDD and ΔLVEF in non-responders and responders.**Data are presented using regression coefficient (β) with 95% confidence interval [CI]; p-value. Ns indicates non-significant. DhCer, dihydroceramide; GlcCer, glucosylceramide; dhSM, dihydrosphingomyelin, PC, phosphatidylcholine; DAG, diacylglycerol; LVEDD, left ventricular end-diastolic diameter; LVEF, left ventricular ejection fraction; TAG, triacylglycerol.

**
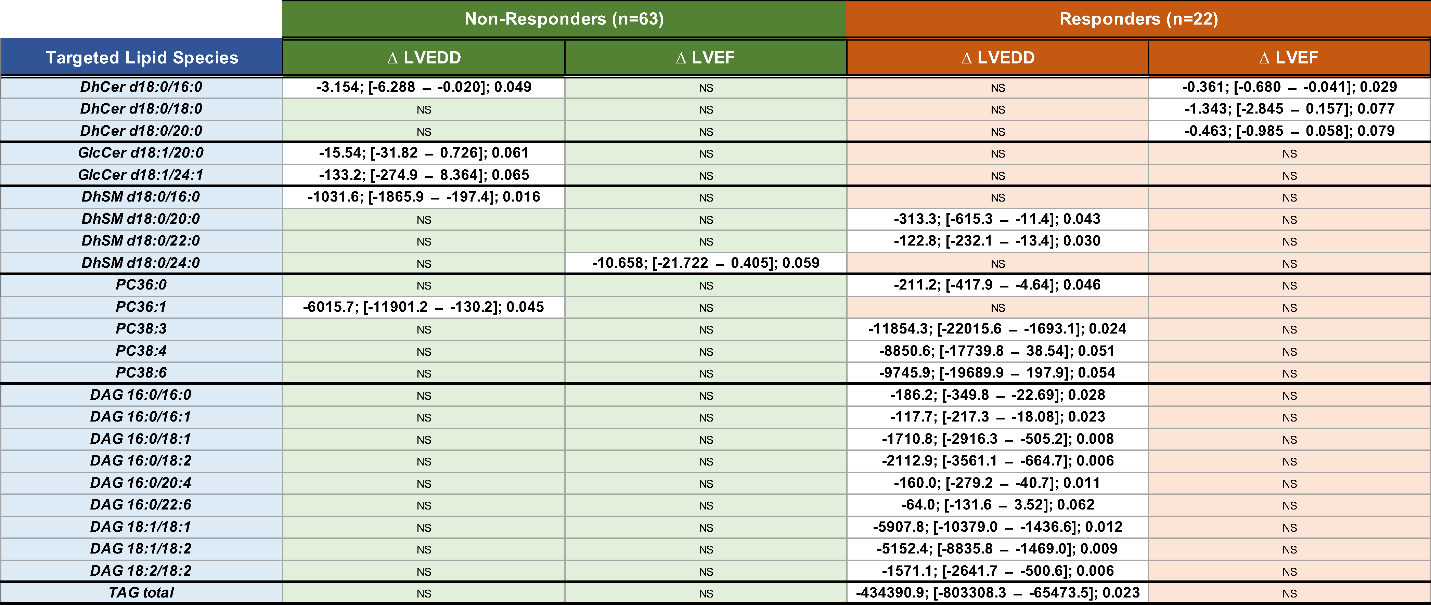
**
